# Genetic analysis and population structure of wild and cultivated wishbone flower (*Torenia fournieri* Lind.) lines related to specific floral color

**DOI:** 10.7717/peerj.11702

**Published:** 2021-07-05

**Authors:** Shikai Guan, Qian Song, Jinye Zhou, Haixia Yan, Yuxiang Li, Zibin Zhang, Dayan Tao, Shuming Luo, Youqiang Pan

**Affiliations:** 1 Flower Research Institute, Guangxi Academy of Agricultural Sciences, Nanning, Guangxi, China; 2 Guangxi Academy of Agricultural Sciences, Nanning, Guangxi, China; 3 Plant Breeding Institute, Faculty of Agriculture and Environment, University of Sydney, Cobbitty, NSW, Australia; 4 Guangxi Crop Genetic Improvement and Biotechnology Laboratory, Guangxi Academy of Agricultural Sciences, Nanning, Guangxi, China

**Keywords:** Wishbone flowers, Transposon, Genetic diversity, Genetic structure, Plant breeding, iPBS

## Abstract

**Background:**

The wishbone flower or *Torenia fournieri* Lind., an annual from tropical Indochina and southern China, is a popular ornamental plant, and many interspecific (*T*. *fournieri* × *T. concolor*) hybrid lines have been bred for the international market. The cultivated lines show a pattern of genetic similarity that correlates with floral color which informs on future breeding strategies. This study aimed to perform genetic analysis and population structure of cultivated hybrid lines comparing with closely related *T. concolor* wild populations.

**Methods:**

We applied the retrotransposon based iPBS marker system for genotyping of a total of 136 accessions from 17 lines/populations of *Torenia*. These included 15 cultivated lines of three series: Duchess (A, B, C); Kauai (D, E, F, G, H, I, J); Little Kiss (K, L, M, N, P) and two wild *T. concolor* populations (Q and R). PCR products from each individual were applied to estimate the genetic diversity and differentiation between lines/populations.

**Results:**

Genotyping results showed a pattern of genetic variation differentiating the 17 lines/populations characterized by their specific floral colors. The final PCoA analysis, phylogenetic tree construction, and Bayesian population structural bar plot all showed a clear subdivision of lines/populations analysed. The 15 cultivated hybrid lines and the wild population Q that collected from a small area showed the lowest genetic variability while the other wild population R which sampled from a larger area had the highest genetic variability.

**Discussion:**

The extremely low genetic variability of 15 cultivated lines indicated that individual line has similar reduction in diversity/heterozygosity from a bottleneck event, and each retained a similar (but different from each other) content of the wild genetic diversity. The genetic variance for the two wild *T. concolor* populations could be due to our varied sampling methods. The two wild populations (Q, R) and the cultivated hybrid lines (I, K, M, N, P) are genetically more closely related, but strong positive correlations presented in cultivated lines A, C, E, M, and N. These results could be used to guide future *Torenia* breeding.

**Conclusions:**

The genetic variation and population structure found in our study showed that cultivated hybrid lines had similar reduction in diversity/heterozygosity from a bottleneck event and each line retained a similar (but different from each other) content of the wild genetic diversity, especially when strong phenotypic selection of floral color overlaps. Generally, environmental factors could induce transposon activation and generate genetic variability which enabled the acceleration of the evolutionary process of wild *Torenia* species. Our study revealed that wild *Torenia* populations sampled from broad geographic region represent stronger species strength with outstanding genetic diversity, but selective breeding targeting a specific floral color decreased such genetic variability.

## Introduction

The genus *Torenia* belongs to the class Magnoliopsida, order Scrophulariales and family Scrophulariaceae. There are more than 50 species native to tropical and subtropical Asia and Africa (Yamazaki, 1985; Hsieh & Yang, 2002). According to Yamazaki (1985), the significant biodiversity regions for this genus have been found in Southeast Asia (19 in Thailand, 20 in Cambodia, Laos, and Vietnam) and southern China (Hsieh & Yang, 2002). Wishbone flower or *Torenia* is the common name for several species in the genus including *Torenia fournieri* Lind., *T. concolor* Lindl., *T. asiatica* L. and *T. hybrida* (*T. fournieri* × *T. concolor*) (OGTR, 2008). *T. fournieri*, an annual from tropical Indochina, is one of the most important species in the genus for ornamental use in tropical and subtropical countries, especially as a bedding plant during summer in temperate regions. An erect type cultivar of this particular species with a violet floral color was initially commercialized. Then in 1988, PanAmerican Seed (West Chicago, IL, USA) released the Crown series with pink, white, and reddish-purple color lines. Selected from an interspecific cross between *T. fournieri* and *T. concolor* in 1995, a trailing type cultivar called the Summer Wave series was later released by Suntory Ltd. (Osaka, Japan). For more than 30 years, many hybrids have been released as patents or trademark series with a full range of floral colors from white with yellow throats to blue, cobalt, lavender, and violet, making *Torenia* a popular ornamental plant on the international market (Nagase, 1997; Miyazaki & Ohsumi, 1997; Tamura & Miyazaki, 1999; Miyazaki, 1999, 2001, 2002, 2003; Suzuki et al., 2002; Iwaki, 2005). As a consequence, a thorough knowledge of the genetic background of both wild and cultivated *Torenia* could aid future selective breeding of this important genus.

Early research employed *T. fournieri* as an ideal model plant for micromanipulation of artificial fertilization. Two major characteristics of the *Torenia* species included the exceptional protruding embryo sac (Higashiyama et al., 1997) and the synergid cells with polypeptides secreted as pollen tube attractants during fertilization (Okuda et al., 2009) that enable the detailed observation of the fertilization process. Consequently, the micromanipulation of artificial fertilization on *Torenia* as originally developed by Keijzer, Reinders & Leferink-ten (1988) resulted some interspecific hybrids, has since been adopted in a wide range of practical plant gene biotechnological experimentations (Higashiyama et al., 1998, 2001, 2006; Wu, Qin & Zhao, 2008; Wu et al., 2008; Nakamura et al., 2010; Nishihara et al., 2018; Sasaki & Ohtsubo, 2020). However, the resulted interspecific hybrids require genetic analysis.

In relation to new variants, Tandon & Bhutani (1965) first reported the morphological and cytological descriptions of the colchicine-induced *T. fournieri* tetraploid plant, Kikuchi et al. (2005) later undertook cytogenetic analysis with the formation of *Torenia* interspecific hybrids. Kikuchi et al. (2007a) conducted a thorough evaluation of pollen tube growth in cross combinations between *T. fournieri* and 14 related species and some interspecific hybrids were attained. Specifically on the *T. fournieri* and *T. baillonii* hybrid, a wide range of publications relating to cytological variations, genome size, interspecific genomic affinity, karyotypic variation, centromere separation and associations during mitosis or meiosis occurred (Kikuchi et al., 2006; Kikuchi et al., 2007b; Nuntha et al., 2016; Nuntha et al., 2017). Wei (2010) completed some experimentation on inheritance of the floral color, and analyzed the barriers to distant hybridization between closely related species in the genus *Torenia*, whilst Liang (2014) outlined a practical method on selective and mutational breeding to further achieve interspecific polyploid hybrids. The most recent interspecific *Torenia* hybrid was obtained through ovule culture (Laojunta et al., 2019). However, there has been no population genetic analysis on interspecific hybrids of *Torenia*.

A significant issue in the commercialization of *Torenia* interspecific hybrids is floral color, and specific colors have been released as individual cultivars. Some early chemo-genetic investigations of the floral color of *T. fournieri* revealed the inheritance pattern of anthocyanin concentrations in their flowers, and similarly, the inheritance pattern of the carotenoids in the petals of *T. baillonii* and *T. fournieri* have been described (Endo, 1962; Lang, 1970; Hess, 1971). Suzuki et al. (2000) reported that hybrid floral color in *Torenia* could be modified by suppressing the genes for anthocyanin biosynthesis whilst such floral color was later shown to be controlled, expressed and modified by the flavonoid 3′-hydroxylase and flavone synthase II genes (Ueyama et al., 2002). Through RNAi suppression of the anthocyanidin synthase gene, Nakamura et al. (2006) achieved pure white flowers with a higher frequency and better stability than through the antisense and sense suppression method. Furthermore, the floral pigmentation in *Torenia* species could be mutated by heavy ion beam irradiation (Miyazaki et al., 2006). Additionally, *Torenia* species exhibited a high degree of transformation by way of the *Agrobacterium* system (Aida & Shibata, 1995), making them popular for transgenic studies (Aida et al., 2000a, 2000b; Aida, 2008; Nishihara et al., 2013; Masahiro et al., 2014). These molecular biotechnological research results significantly enhanced our knowledge about floral color gene functioning in the *Torenia* genus. Consequently, these research findings provide useful background information for the future plant genetic improvement of *Torenia* floral color in cultivated and wild populations.

Regarding genetic activity and homeotic genes in particular, we know that they can regulate the development of anatomical structures in various organisms during evolution. Bradley et al. (1993) reported that some complementary floral homeotic phenotypes resulted from the opposite orientations of a transposon at the *plena* locus. In addition, the first double flower phenotype in Japanese morning glory (*Ipomoea nil*) resulted from gene duplication through insertion of an En/Spm-related transposable element into a floral homeotic gene (Nitasaka, 2003). Similarly, the chimeric TCP3 repressor in *Arabidopsis* was also found to be responsible for novel floral traits in *T. fournieri* and *Chrysanthemum morifolium* (Narumi et al., 2011). For floral gene expression in *T. fournieri*, the novel paracorollas trait has been generated when the floral homeotic genes were induced by the plant hormone forchlorfenuron (Niki et al., 2012) because the relative cytokinin biosynthesis system promoted a triggering expression of the cytokinin oxidase/dehydrogenase gene during such treatments (Niki et al., 2013a; Niki et al., 2013b). Such research findings enable us to conclude that some transposons in the homeotic genes can be the major genetic factors regulating certain phenotypic traits of the flowers. Therefore, use of transposon marker systems could be valuable in facilitating the population genetic analysis of *Torenia* in cultivated and wild populations so as to understand their evolutionary history as well as the selective pressure due to breeding for floral colors, in particular.

Specifically, for a practical transposon marker system, Kalendar et al. (2010) designed the iPBS (inter-Primer Binding Site) to deal with the “jumping genes” or transposable sequences of DNA that move from one location to another in the genome. This molecular marker technology utilized the universal presence of a tRNA complement as a reverse transcriptase primer binding site in LTR (Long Terminal Repeat) retrotransposons (Alzohairy et al., 2014). Different to the codominant marker systems such as microsatellite, iPBS is a dominant marker system that requires no previous knowledge of the genome and it is a more powerful DNA genotyping technique than the transposon-based markers established previously (Kalendar, Amenov & Daniyarov, 2019; Androsiuk et al., 2019). This marker system has been successfully applied to a wide range of plants such as ornamentals (Luo et al., 2015; Zhou et al., 2021), fruits (Baránek et al., 2012; Guo et al., 2014; Mehmood et al., 2016; Al-Najm et al., 2016), medicinal plants (Chen & Liu, 2014; Borna et al., 2017), and other different species (Singha, Nandhaa & Singha, 2017; Androsiuk et al., 2019; Androsiuk et al., 2020). Using such a retrotransposon-based iPBS technique, our objectives were to investigate the genetic pattern, population structure and genetic relationships among 136 *Torenia* accessions including 15 cultivated lines from three commercial series (Duchess, Kauai, Little Kiss) and two wild *T. concolor* populations sampled from two different sizes of area. Our overall aim was: (i) to estimate the genetic variation and population structure for *T. fournieri* cultivated lines which could be influenced by breeding selection; (ii) to explain the variation of the genetic diversity between the two wild populations could be differed from the sampling method we used; (iii) to provide insight into the effect on genetic variation in a population experiencing a bottleneck in which strong phenotypic selection overlaps; (iv) to provide a practical model for the genetic analysis of *Torenia* populations so as to better enable selective breeding in the future.

## Materials and Methods

### Material

**Figure 1:**
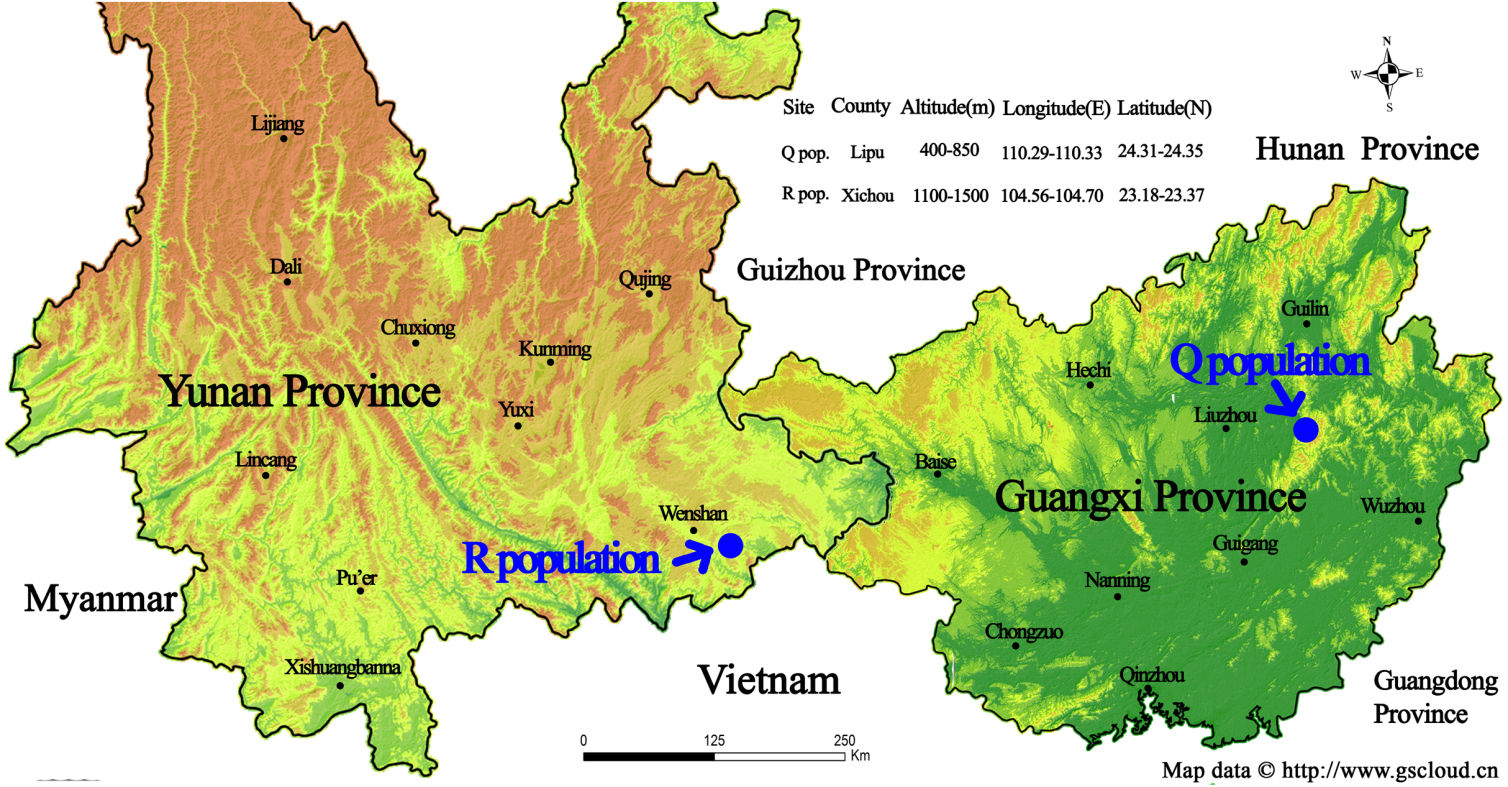
Geographic location of the sampling sites for two wild *Torenia concolor* populations in southern China. This map is provided by Geospatial Data Cloud site, Computer Network Information Center, Chinese Academy of Sciences. (http://www.gscloud.cn)

**Figure 2:**
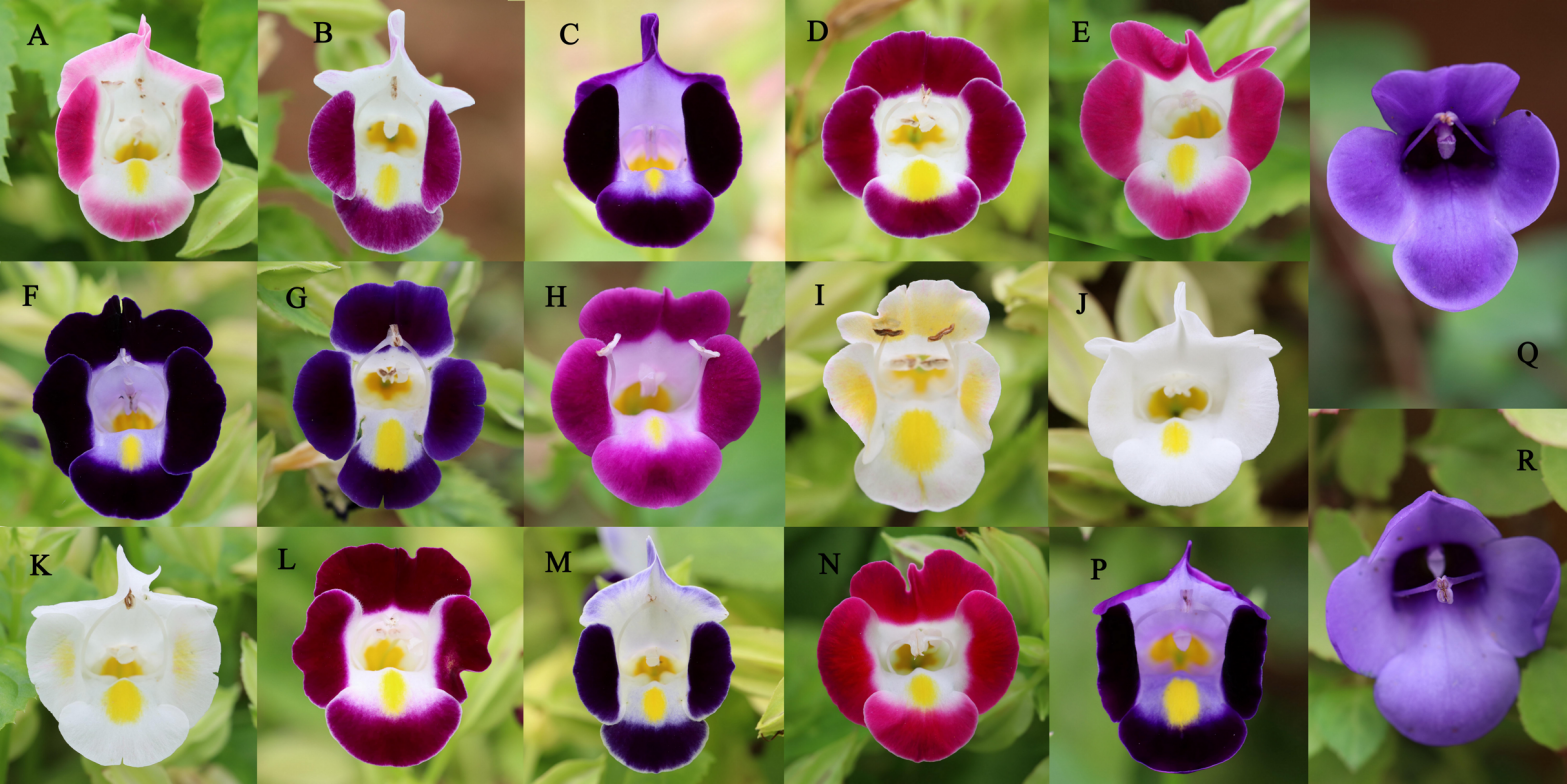
The floral colors for 17 *Torenia* lines/populations. (A) Duchess pink. (B) Duchess burgundy. (C) Duchess deep blue. (D) Kauai burgundy. (E) Kauai rose. (F) Kauai deep blue. (G) Kauai blue and white. (H) Kauai magenta. (I) Kauai lemon drop. (J) Kauai white. (K) Little kiss white. (L) Little kiss burgundy. (M) Little kiss blue and white. (N) Little kiss rose picotee. (P) Little kiss blue. (Q) Wild, blue. (R) Wild, blue.

The list of our 17 lines/populations included A (Duchess: Pink), B (Duchess: Burgundy), C (Duchess: Deep Blue), D (Kauai: Burgundy), E (Kauai: Rose), F (Kauai: Deep Blue), G (Kauai: Blue and White), H (Kauai: Magenta), I (Kauai: Lemon Drop), J (Kauai: White), K (Little Kiss: White), L (Little Kiss: Burgundy), M (Little Kiss: Blue and White), N (Little Kiss: Rose Picotee), P (Little Kiss: Blue), Q (wild: Lipu) and R (wild: Xichou). The origin of the plant materials used in this study are listed in File S1. We purchased a total of 15 *T. hybrida* seed lines from Shanghai Yuanyi Seedling Co. Ltd. These 15 colorful seed lines included three commercial series. Two wild *T. concolor* populations (Q: Lipu and R: Xichou) were collected from two different subtropical habitats in southern China, Lipu in Guangxi Zhuang Autonomous Region and Xichou in Yunnan Province (Fig. 1). To represent all the commercial lines that have been produced by selective breeding and currently released on the international market, approximately 200 seedlings for each line were germinated first, and eight plants were randomly selected to form a line. According to the availability of individual *T. concolor* plant in natural habitats, eight individual accessions of the population Q were collected from a small area with a minimum distance of 50 m, whilst population R was selected across a larger area with a minimum distance of 100 m. Commercial seeds were germinated and grown in a glasshouse under a controlled environment at the Flower Research Institute, Guangxi Academy of Agricultural Sciences in 2019. The floral colors for 17 populations were presented in Fig. 2. With a total of 136 accessions, 15 cultivated lines and two *T. concolor* wild populations were then used for iPBS analysis.

### DNA extraction and quantification

About 100 mg of fresh young leaves from each accession were used for DNA isolation. Genomic DNA for each plant was extracted using the EasyPure^R^ Plant Genomic DNA Kit (TransGen Biotech, Beijing, China) in accordance with the manufacturer’s protocols. The quality and quantity of DNA examined using 2.0% agarose gel electrophoresis by comparison with the standard λ DNA concentrations. A portion of the isolated DNA was diluted with distilled water to 1 ng μL^−1^ concentration and used as the template for later PCR experimentation.

### Protocols for iPBS genotyping

According to the sequence designed by Kalendar et al. (2010), the iPBS primers were synthesized from Sangon Biotech (Songjiang, Shanghai, China) and DNA amplification in our study was conducted using a slightly modified protocol. Using varied annealing temperatures, a total of 40 primers were initially screened with two DNA samples (A1, Q121), and the method was similar to Zhou et al. (2021). Band pattern reproducibility of the PCR amplification was tested by comparison with randomly selected electrophoretic profiles. To confirm band pattern consistency, PCR was performed three times for each primer. Seven polymorphic primers with repeatable and clearly identifiable bands were chosen for the final PCR amplifications (Table 1).

**Table 1:** Seven iPBS primers used in the detection of polymorphism among 136 accessions from 17 *Torenia* lines/populations.

iPBS primer	Sequence (5′–3′)	Ta °C	Number of bands*	Duchess Series	Kauai Series	Little Kiss Series	Wild population	Mean PIC value^1^
2,076	GCTCCGATGCCA	39	21	12	17	14	15	0.249
2,077	CTCACGATGCCA	39	19	12	14	14	15	0.220
2,272	GGCTCAGATGCCA	39	20	15	15	14	15	0.291
2,277	GGCGATGATACCA	43.5	28	22	24	19	18	0.327
2,377	ACGAAGGGACCA	43.5	16	10	11	11	12	0.246
2,387	GCGCCATACCCA	39	20	14	16	13	15	0.300
2,383	GCATGGCCTCCA	43.5	24	14	16	14	18	0.239
Total			148	99	113	99	108	0.267

**Notes:**

* Total accountable bands consistently appearing in two or three repeated experiments.

1 PIC value is calculated as PIC = 1 – [f^2^ + (1 − f)^2^], where f is the frequency of the dominant marker in the data set.

A total of 136 accessions for 17 *Torenia* lines/populations included Duchess series (three population of 24 accessions), Kauai series (seven population of 56 accessions), Little Kiss series (five population of 40 accessions) and wild population (two population of 16 accessions).

The PCR experiments were performed in 20 μL reaction mixtures containing 1 μL (1 ng) genomic DNA, 10 μL 2 × Bench Top Taq Master Mix (Biomiga, San Diego, CA, USA), 1 μL of 10 μM primer (single primer), and 8 μL of ddH_2_O. The PCR profile included an initial hot start at 95 °C for 3 min, 40 cycles of denaturation at 94 °C for 30 s, annealing at 39–43.5 °C for 30 s and with an extension at 72 °C for 2 min. There was a final extension at 72 °C for 5 min and the program was terminated by holding at 4 °C. Using 0.2 mL tubes or 96-well plates, all the reactions was performed in a LIFE ECO Thermal Cycler (Bori, Hangzhou, China). Final PCR products were electrophoresed at 70 V for 3.5 h with 1.5% (w/v) agarose gel in 1 × TAE buffer (0.04 M Tris-acetate, 0.001 M EDTA). The standard Dongsheng LD DS5,000 ladder (Guangzhou, China) was used as the marker to estimate fragment lengths. GoldView Ⅰ Nuclear Acid Dye (10,000×) (Suobao, Shanghai, China) was used to post-stain the gels for 20–30 min and photographs were taken using the GenoSens 1860 model with Clinx GenoSens Capture Software (Qinxiang, Shanghai, China).

### Scoring and analyzing the iPBS data

For the process of scoring, the DNA bands were carefully sized, visually examined and all faint bands were excluded. Only clear bands were included and same sized bands were treated as a single locus. The data record for each locus used ‘1’ for presence of a band and ‘0’ for absence so as to form a binary matrix. The polymorphic informative content (PIC) value for each primer was calculated by PIC = 1 − [f^2^ + (1 − f)^2^], where f is the frequency of the dominant marker in the data set. Accountable bands consistently appeared in all the repeated experiments.

**Figure 3:**
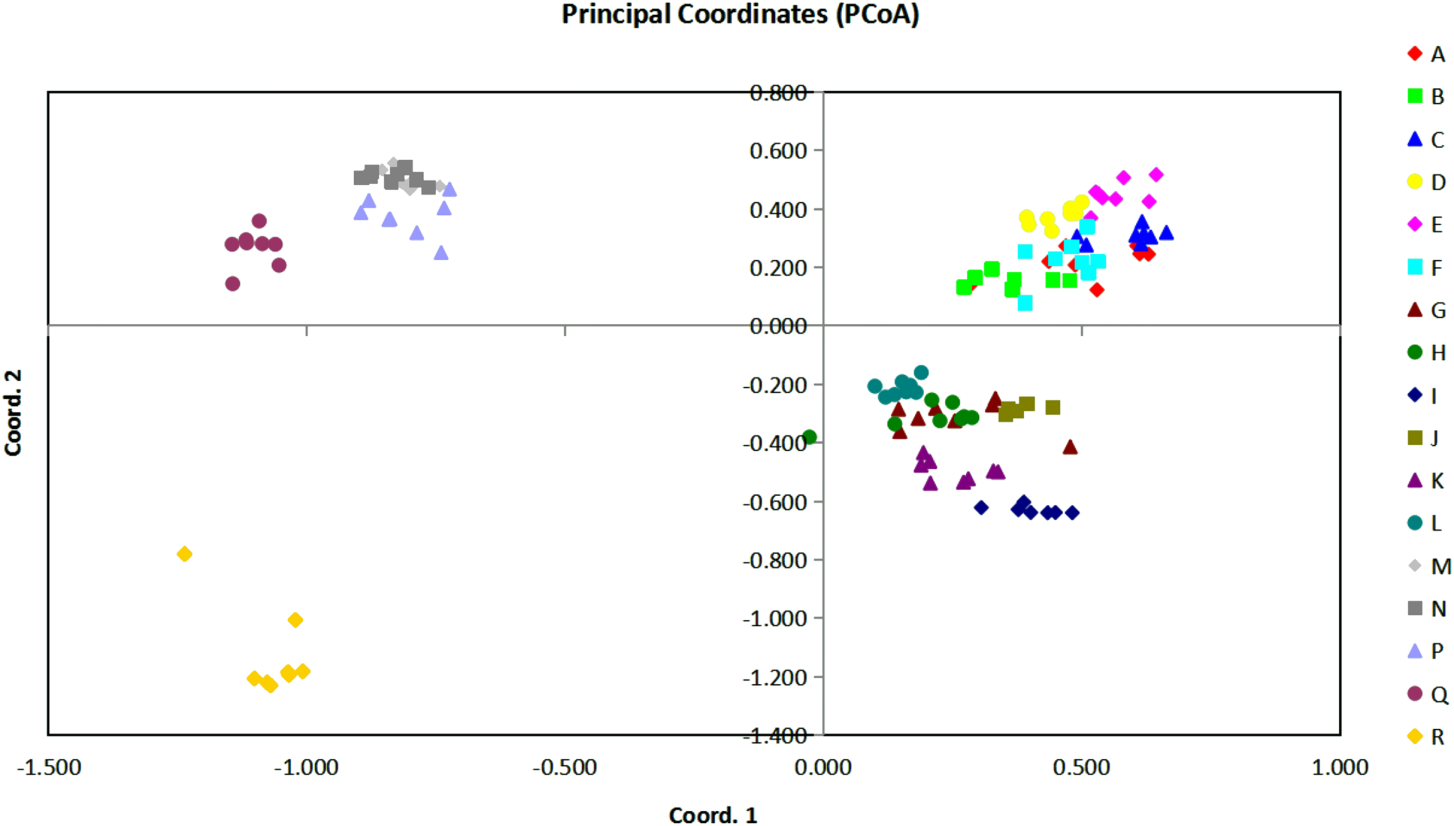
The plot of Coordinate 1 vs Coordinate 2 in principal coordinate analysis (PCoA) using all the Euclidean distances for the 136 individual accessions of 17 *Torenia* lines/populations.

Accessions with different names that were completely matched at all the loci were considered as duplicates. Duplicates in the data set were checked by multi-locus matching. All analyzes were performed with the GenAIEx 6.5 program (Peakall & Smouse, 2012) (Fig. 3). Pair-wise genetic distance was first calculated using the DISTANCE procedure. Later analysis included principal coordinates analysis (PCoA), and data were collected also as previously described in Peakall & Smouse (2012). The individual based pairwise distance matrices for populations were also used for hierarchical AMOVA analysis, and pairwise PhiPT values from the Phi-statistics were used to measure population differentiation due to genetic structure estimated from the genetic polymorphism data. Each time, the data were subjected to 1,000 permutations. As an indication of the relative contribution of population differentiation, the greater the pairwise PhiPT values mean the greater the differences between populations. The total genetic variation was partitioned across three levels: within populations (Phi-PT), among populations within taxa (Phi-PR), and among taxa (Phi-RT).

**Figure 4:**
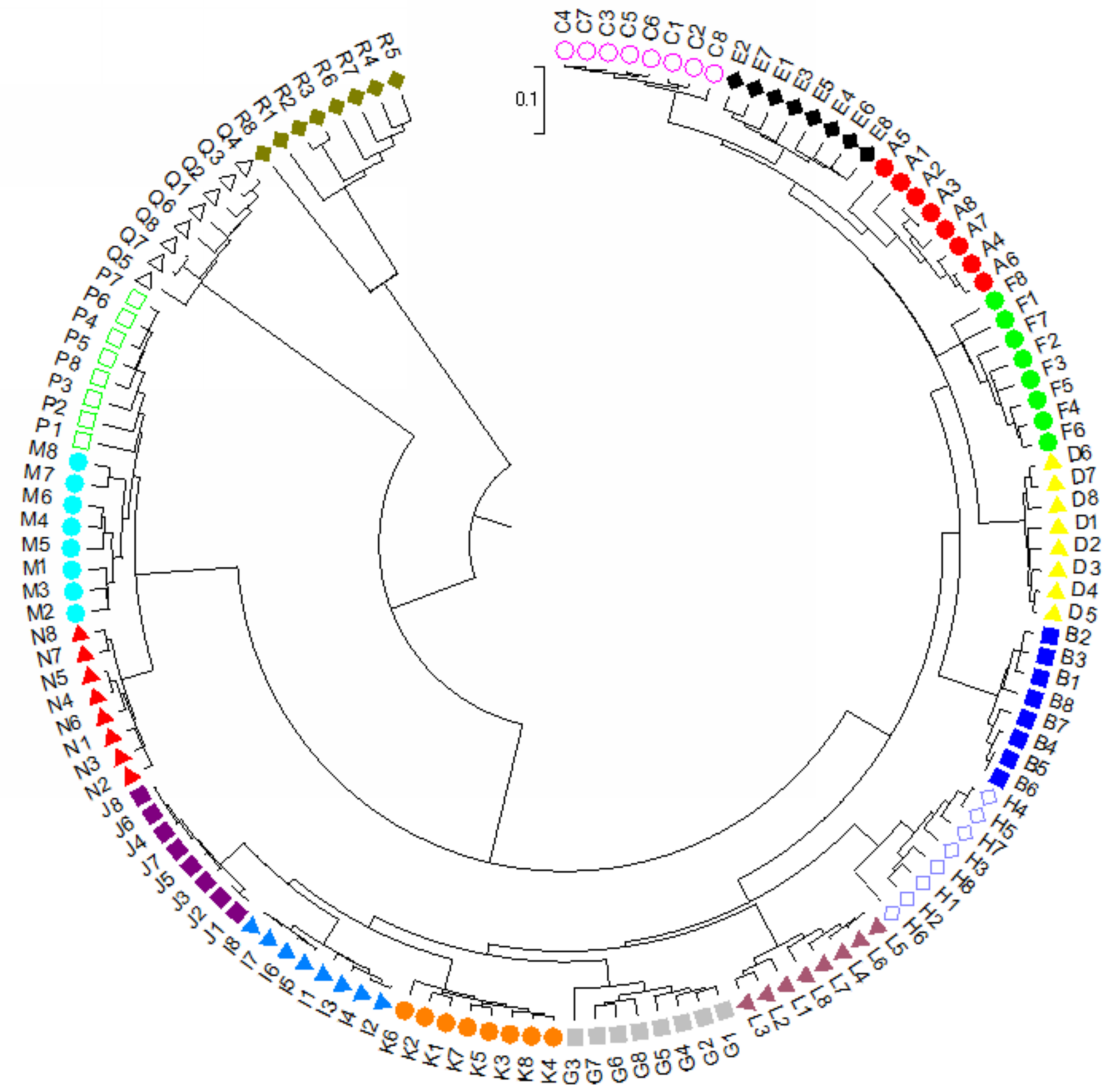
The evolutionary relationship of 136 *Torenia* taxa in 17 lines/populations derived from the combined data of seven iPBS primers. A, Duchess pink; B, Duchess burgundy; C, Duchess deep blue; D, Kauai burgundy; E, Kauai rose; F, Kauai deep blue; G, Kauai blue and white; H, Kauai magenta; I, Kauai lemon drop; J, Kauai white; K, Little kiss white; L, Little kiss burgundy; M, Little kiss blue and white; N, Little kiss rose picotee; P, Little kiss blue; Q, Wild, blue; R, Wild, blue.

For dendrogram construction, data were collected and used the MEGA7.0.14 software (Kumar, Stecher & Tamura, 2016) for analysis as previously described in Zhou et al. (2021). There was a total of 148 positions for our 136 taxa in the final dataset. Using the UPGMA method (Sneath & Sokal, 1973), the optimal tree with the sum of branch length = 5.0639 is shown. The final tree is drawn to scale, with branch lengths in the same units as those of the evolutionary distances used to infer the phylogenetic dendrogram. The evolutionary distances in the optimal tree were computed and are in the units of the number of base substitutions per site (Tamura, Nei & Kumar, 2004). A final dendrogram revealing the evolutionary relationship of 136 *Torenia* taxa in 17 lines/populations is listed in Fig. 4.

**Figure 5:**
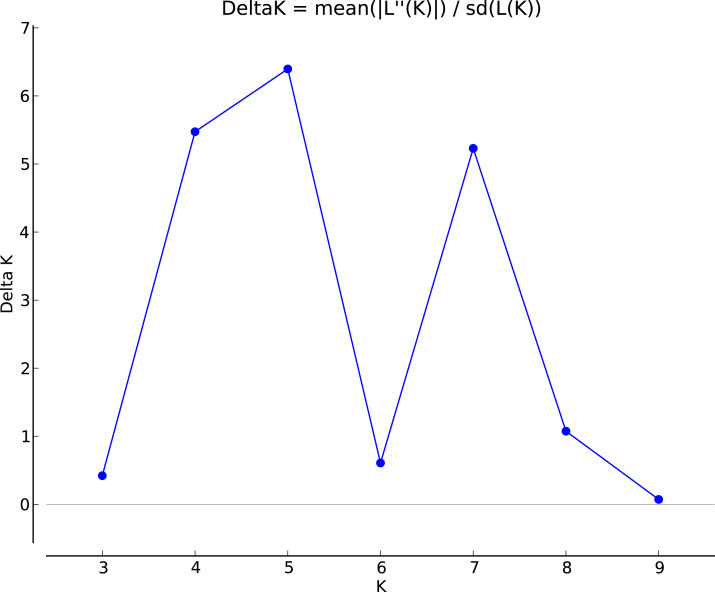
The final hierarchical level of genetic structure for 17 *Torenia* lines/populations using a Bayesian model-based cluster construction based on allelic variations at seven iPBS loci (Earl & Vonholdt, 2012). Plot of the delta K (filled circles, solid line) according to the values of the second-order rate of change of *L*(K), ΔK, and data between successive K values.

**Figure 6:**
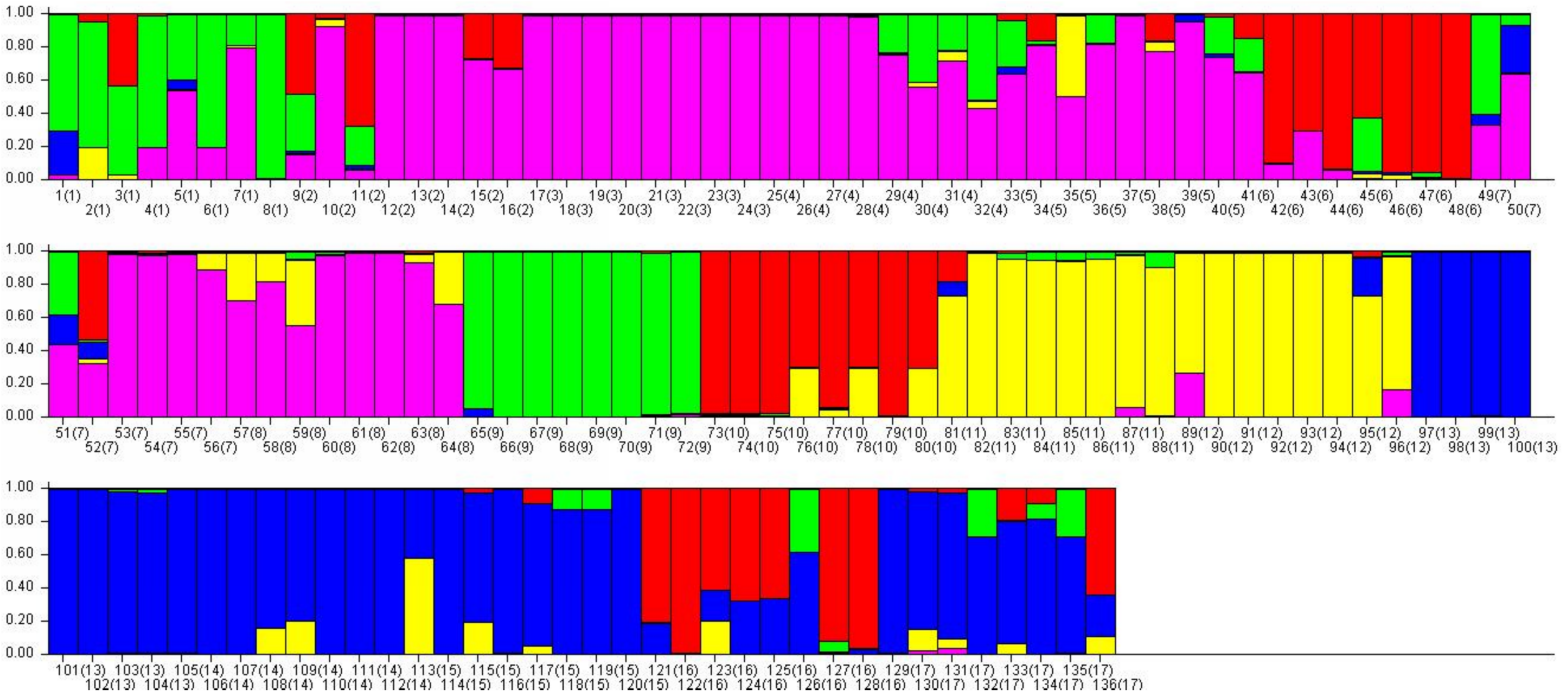
The final hierarchical level of genetic structure for 17 *Torenia* lines/populations using a Bayesian model-based cluster construction based on allelic variations at seven iPBS loci (Earl & Vonholdt, 2012). Bar plots of the population genetic structure achieved at K = 5.

The genotypic data were also analyzed using a Bayesian model-based clustering method implemented through STRUCTURE V2.3.4 (Pritchard, Stephens & Donnelly, 2000) for population structure to classify 17 *Torenia* lines/populations into different groups. Data were collected as previously described in Zhou et al. (2021). In Fig. 5, the optimal number of clusters or the most likely K value (K = 5) was based on Evanno, Regnaut & Goudet (2005). Population structures were constructed using a Bayesian model-based clustering computation from STRUCTURE HARVESTER (Earl & Vonholdt, 2012), and a final population structure was optimized in Fig. 6.

## Results

### Genetic DNA polymorphism

We screened seven primers with high polymorphism from 40 iPBS primers. These seven primers (2,076, 2,077, 2,272, 2,277, 2,377, 2,387 and 2,383) were subsequently selected for the final PCR amplifications (Table 1). The sizes of scorable and reproducible bands from all these seven primers ranged from 150 to 4,500 bp. The number of unique banding patterns among these 136 accessions validated the use of this retrotransposon based iPBS marker system for individual identification of the 17 lines/populations tested.

A total of 148 scorable bands were amplified using the seven primers selected, and they revealed a high degree of genetic variability within each lines/population and among lines/populations. Detailed results for these seven primers including the sequence of the primer, the annealing temperature used in the PCR, number of amplified bands, number of bands specific for three of the commercial series (Duchess, Kauai, Little Kiss) and wild populations, and mean PIC values are presented in Table 1. The highest number of bands (28) was produced by primer 2,277 while primer 2,377 generated the lowest (16). Primer 2,277 had the highest PIC value (0.327) whereas primer 2,077 had the lowest (0.220). The mean PIC value for these seven primers was 0.267. For dominant markers, the PIC values varied between 0 to 0.5, and any values more than 0.25 were classified as highly informative. These results indicate that a wide range of genomic DNA diversity existed across the 17 *Torenia* lines/populations tested.

Our results also showed that a range of different private bands were specifically amplified for lines/populations with varied floral colors. As for the two wild populations, population Q shared more common bands with the 15 cultivated lines, while population R had the highest number of specific bands. For the 15 cultivated lines, there were a total of 152 population-specific bands from seven primers. Primer 2076 represented 33 specific bands (in red and blue). Primer 2,277 showed a maximum of 36 while primer 2,077 had a minimum of six (File S5).

### Heterozygosity for 17 *Torenia* lines/populations

Summary statistics including number of different alleles, number of effective alleles, Shannon’s information index, expected heterozygosity, unbiased expected heterozygosity, and percentage of the polymorphic loci are presented in Table 2. The number of different alleles (Na) ranged from 0.4865 (J) to 0.9932 (R), with an average of 0.6586. The number of effective alleles (Ne) varied from 1.0243 (J) to 1.2885 (R), with an average of 1.0899. The Shannon’s information index for the 17 lines/populations ranged from 0.0200 (J) to 0.2453 (R), with an average of 0.0758. The expected heterozygosity values (He) fluctuated from 0.0138 (J) to 0.1655 (R), with an average of 0.0513. The unbiased expected heterozygosity (uHe) ranged from 0.0147 (J) to 0.1766 (R) with an average of 0.0547 whereas the percentage of polymorphic loci ranged from 3.38% (J) to 45.27% (R). Therefore, the most variation of genetic diversity existed between the cultivated line J and the wild population R.

**Table 2:** Summary statistics of 136 *Torenia* accessions from 17 lines/populations evaluated with seven iPBS primers.

Populations	*N*	Na	Ne	I	He	uHe	Polymorphic loci %
A	8	0.7500 ± 0.0598	1.1101 ± 0.0223	0.0928 ± 0.0178	0.0629 ± 0.0123	0.0671 ± 0.0131	16.89%
B	8	0.6757 ± 0.0518	1.0514 ± 0.0153	0.0458 ± 0.0127	0.0305 ± 0.0087	0.0325 ± 0.0093	8.78%
C	8	0.6351 ± 0.0481	1.0302 ± 0.0121	0.0269 ± 0.0099	0.0178 ± 0.0067	0.0190 ± 0.0072	5.41%
D	8	0.6892 ± 0.0524	1.0567 ± 0.0168	0.0486 ± 0.0132	0.0325 ±0.0091	0.0346 ± 0.0097	9.46%
E	8	0.7568 ± 0.0564	1.0894 ± 0.0202	0.0770 ± 0.0163	0.0518 ± 0.0112	0.0553 ± 0.0119	14.19%
F	8	0.7365 ± 0.0609	1.1196 ± 0.0236	0.0990 ± 0.0184	0.0675 ± 0.0128	0.0720 ± 0.0136	17.57%
G	8	0.7027 ± 0.0595	1.0973 ± 0.0208	0.0842 ± 0.0169	0.0566 ± 0.0116	0.0604 ± 0.0124	15.54%
H	8	0.7365 ± 0.0609	1.1305 ±0.0248	0.1047 ± 0.0192	0.0723 ± 0.0134	0.0772 ± 0.0143	17.57%
I	8	0.5338 ± 0.0493	1.0309 ± 0.0122	0.0274 ± 0.0100	0.0182 ± 0.0069	0.0194 ± 0.0073	5.41%
J	8	0.4865 ± 0.0465	1.0243 ± 0.0112	0.0200 ± 0.0090	0.0138 ± 0.0062	0.0147 ± 0.0066	3.38%
K	8	0.5473 ± 0.0528	1.0464 ± 0.0149	0.0409 ± 0.0121	0.0271 ± 0.0083	0.0290 ± 0.0088	8.11%
L	8	0.6216 ± 0.0562	1.0600 ± 0.0164	0.0549 ± 0.0135	0.0360 ± 0.0092	0.0383 ± 0.0098	11.49%
M	8	0.6081 ± 0.0611	1.1151 ± 0.0234	0.0934 ± 0.0182	0.0644 ± 0.0127	0.0686 ± 0.0135	15.54%
N	8	0.4932 ± 0.0530	1.0609 ± 0.0186	0.0472 ± 0.0137	0.0327 ± 0.0097	0.0349 ± 0.0103	8.11%
P	8	0.6284 ± 0.0616	1.1111 ± 0.0232	0.0908 ± 0.0178	0.0620 ± 0.0125	0.0662 ± 0.0133	16.22%
Q	8	0.6014 ± 0.0619	1.1067 ± 0.0222	0.0900 ± 0.0175	0.0609 ± 0.0121	0.0650 ± 0.0129	16.22%
R	8	0.9932 ± 0.0788	1.2885 ±0.0309	0.2453 ± 0.0239	0.1655 ± 0.0167	0.1766 ± 0.0178	45.27%
**Mean**	8	0.6586 ± 0.0141	1.0899 ± 0.0050	0.0758 ± 0.0039	0.0513 ± 0.0027	0.0547 ± 0.0029	13.83% ± 2.28%

**Notes:**

*N*, number of sample size; Na, number of different alleles; Ne, number of effective alleles = 1/(p ^ 2 + q ^ 2); I, Shannon’s information index = −1* (p * Ln (p) + q * Ln(q)); He, expected heterozygosity = 2 * p * q; uHe, unbiased expected heterozygosity = (2N/(2N − 1))* He. Where for diploid binary data and assuming Hardy-Weinberg Equilibrium, q = (1 − Band Freq.) ^ 0.5 and *p* = 1 − q.

A, Duchess Pink; B, Duchess Burgundy; C, Duchess Deep Blue; D, Kauai Burgundy; E, Kauai Rose; F, Kauai Deep Blue; G, Kauai Blue and White; H, Kauai Magenta; I, Kauai Lemon Drop; J, Kauai White; K, Little Kiss White; L, Little Kiss Burgundy; M, Little Kiss Blue and White; N, Little Kiss Rose Picotee; P, Little Kiss Blue; Q, Lipu; R, Xichou.

In Table 3, pairwise population PhiPT values for a total of 136 accessions from 17 lines/populations are listed. The greatest pairwise population PhiPT values are indicated in bold, and their values varied from N/I (0.917) to Q/I (0.898). These results indicate that cultivated line N and wild population Q are considerably different or varied compared to lines C, I and J. In addition, the smallest pairwise population PhiPT values are marked in red and these values were separated in two clusters. The first group contained M/P (0.336), N/P (0.562), M/N (0.588), indicating less differentiation among populations M, N, and P. The second group included A/E (0.542), A/F (0.563), F/E (0.581), and these results showed relatively close genetic relationships among populations A, E, and F. Comparing all the 15 cultivated lines with the two wild *T. concolor* populations in File S2, the generalized genetic distance, and relationships within and among populations outlined an overall population genetic evolutionary history. Our AMOVA analysis further showed that most of the described genetic variation for 17 lines/populations occurred among lines/populations (79%), and the variation within lines/populations accounted for the remaining 21%. However, the variation among 15 cultivated lines was 14%, and the variation within lines was 86% (Table 4).

**Table 3:** Pairwise population PhiPT values for a total of 136 accessions from 17 *Torenia* lines/populations.

	A	B	C	D	E	F	G	H	I	J	K	L	M	N	P	Q	R
A	0.000																
B	0.700	0.000															
C	0.640	0.839	0.000														
D	0.686	0.769	0.798	0.000													
E	0.542	0.752	0.638	0.652	0.000												
F	0.563	0.722	0.702	0.652	0.581	0.000											
G	0.700	0.779	0.800	0.802	0.738	0.699	0.000										
H	0.673	0.747	0.806	0.758	0.746	0.728	0.621	0.000									
I	0.784	0.871	0.890	0.878	0.831	0.807	0.690	0.752	0.000								
J	0.754	0.852	0.895	0.875	0.809	0.757	0.740	0.765	0.830	0.000							
K	0.751	0.855	0.878	0.865	0.806	0.779	0.710	0.708	0.771	0.801	0.000						
L	0.709	0.793	0.856	0.827	0.758	0.739	0.701	0.695	0.827	0.802	0.730	0.000					
M	0.762	0.805	0.848	0.821	0.790	0.772	0.768	0.760	0.858	0.842	0.812	0.779	0.000				
N	0.829	0.877	**0.913**	0.878	0.840	0.835	0.835	0.813	**0.917**	**0.912**	0.878	0.843	0.588	0.000			
P	0.767	0.817	0.851	0.827	0.796	0.784	0.765	0.749	0.851	0.838	0.808	0.783	0.336	0.562	0.000		
Q	0.849	0.880	**0.901**	0.878	0.854	0.841	0.843	0.848	**0.898**	**0.904**	0.887	0.863	0.797	0.842	0.806	0.000	
R	0.758	0.770	0.810	0.777	0.778	0.746	0.750	0.725	0.779	0.791	0.768	0.752	0.716	0.759	0.705	0.754	0.000

**Notes:**

Pairwise population PhiPT values below diagonal. Values more than 0.895 marked as bold and values less than 0.600 are indicated by a subscript.

A, Duchess Pink; B, Duchess Burgundy; C, Duchess Deep Blue; D, Kauai Burgundy; E, Kauai Rose; F, Kauai Deep Blue; G, Kauai Blue and White; H, Kauai Magenta; I, Kauai Lemon Drop; J, Kauai White; K, Little Kiss White; L, Little Kiss Burgundy; M, Little Kiss Blue and White; N, Little Kiss Rose Picotee; P, Little Kiss Blue; Q, Lipu; R, Xichou.

**Table 4:** Partitioning of genetic diversity for 17 (15) *Torenia* populations by AMOVA [PhiPT = 0.794 (0.140)].

Source of variation	d.f.	Sum of square	Mean squares	Estimated variance	Percentage of total variation
Among populations	16 (16)	2,098.441 (15.375)	131.153 (0.961)	15.879 (0.068)	79% (14%)
Within populations	119 (119)	489.875 (49.750)	4.117 (0.418)	4.117 (0.418)	21% (86%)
Total	135 (135)	2,588.316 (65.125)		19.996 (0.486)	100% (100%)

**Notes:**

d.f. = degree of freedom.

Significance tests (999 permutations); *p* < 0.001. The values in parentheses are result for the 15 cultivated lines.

### Principal coordinates analysis (PCoA) for line/population correlations

Based on all the Euclidean distances between all individual accessions from our 17 *Torenia* line/populations, principal coordinates analysis (PCoA) showed that the value of the first three PCoA axes accounted for a total variation of 45.03% (first axis = 22.22%, second = 12.26%, third = 10.56%) (Fig. 3). The spatial demonstration of the relative genetic distances revealed the pattern of distribution of all the 136 accessions according to their genetic components or background. Projection of the analyzed lines/populations on the first two axes showed that the accessions of lines A–F were mainly clustered on the top right plane whilst the accessions of lines G–L were distributed on the lower right plane. One sub-group including lines M, N, P together with another sub-group of the wild population Q were scattered on the upper left plane. The wild population R was scattered on the lower left plane as the only single population registered there. Among this overall pattern of distribution, accessions of each line/population tended to be clustered together closely, but the position of the accessions for several commercial lines overlapped with a certain percentage.

In relation to the PCoA analysis, a total of 148 loci were combined into eight components as eigen values by axis in relation to the sample eigen vectors and the list of these results are presented in File S3. The first component, which accounted for 51.49% of the total variability, included lines A, C, E with high eigen values (marked in red), and lines/populations R, Q, N with negative eigen values (marked in blue). Thus, lines A, C, E had a high positive correlation with the highest loading factors whilst populations R, Q, N had a high negative correlation with the lowest loading factors. Similarly, for the second component which accounted for 28.40% of the whole variability, lines E, M, N (marked in red) were positively correlated with higher loading factors, but lines R, I, K (marked in blue) were negatively correlated with lower loading factors. The third component accounted for 26.20% of the variability, and lines R, Q, D were positively correlated with higher loading factors whilst K, M, P were negatively correlated with lower loading factors. All the other components were interpreted similarly, and a combined result from all the eight components could be formed to reveal the overall genetic correlation relationships of the 17 *Torenia* lines tested.

### Phylogenetic relationships for 17 *Torenia* lines/populations

Apart from the above principal coordinates analysis (PCoA), the evolutionary genetic relationship of our 136 accessions was also drawn from the combined data from seven iPBS primers (see above) using MEGA7 software (Fig. 3). The phylogenetic dendrogram was constructed to scale with the branch lengths matched to reveal the evolutionary distances. All the 136 accessions from the 17 lines/populations were placed into five major clusters in the final optimal tree (Fig. 4, File S4). The first cluster included all the 48 accessions from six lines/populations A–F. The second group included 48 accessions of six lines G–K. The third group included all of the 24 accessions in lines M, N and P. The fourth cluster included all the eight accessions of the wild population Q. The fifth cluster contained all of the eight accessions of the wild population R. Consequently, each of the first two major clusters in the dendrogram contained several sub-groups. It is noteworthy that all of the 136 accessions from the 17 lines/populations were clearly grouped according to the origin of the line/population with reference to their specific floral color (File S4).

### Population genetic structure and differentiations

Population stratification of the accessions was based on the most probable or maximum number of K because ΔK is the potential number of genetic clusters that may exist in the overall sample of individuals examined. To calculate the value of ΔK and the second-order rate of change of the likelihood function of K and m (|L″(K)|)/sd[L(K)], the optimal number of genetic clusters for these 136 accessions was determined to be five (Kopt = 5, Fig. 5). Consequently, the optimal number of clusters of the uppermost hierarchical level of the population structure was achieved based on K = 5 (Fig. 6). The final genetic structure showed significant differentiation with a continuous linear order of the 136 accessions of the 17 lines/populations with their specific floral color. Within the final bar plot, each colored bar represents the most likely ancestry of the cluster for the whole or part of the genotype derived, and each vertical line represents an individual multi-locus genotype. Thus, multiple-colored individual accessions have an admixture of genotype components from multiple clusters or groups. Results from these overall structural partitions agreed significantly with the distribution pattern plotted in the principal coordinate analysis mentioned above (Fig. 3), and the evolutionary phylogenetic relationships outlined in the dendrogram (Fig. 4).

## Discussion

A collection of 136 accessions of 15 cultivated lines and two wild *T. concolor* populations with specific floral color were investigated using seven retrotransposon-based iPBS primers, and our results are presented as a PCoA plot, a phylogenetic tree, and an optimized population genetic structure bar plot. All the 148 loci generated from seven selected primers were sufficiently powerful to discriminate genetically our individual accessions into the origin of the line/population, whilst indicating an obvious pattern of genetic relationships within and among the lines/populations. Results from these analyses, individually and combined, indicate that some morphological traits such as the floral colors could be used as an associated genotypic identification, thereby potentially guiding breeding practices that target such traits. This research also showed that the comparison of cultivated *T. fournieri* lines and *T. concolor* wild populations are informative, and these two different species are being compared when analyzing the results.

We know that population structure analyses using dominant markers such as the iPBS system can be weakened if the heterozygosity remains undetected or underestimated. To prevent such an issue in our analysis, we adopted *Phi*-statistics which are a modified version of the conventional Wright’s *F*-statistics. Taking into consideration the genealogical information from the molecular data, the pairwise population PhiPT values were applied to measure the reduction of the expected level of heterozygosity in the population compared to a Hardy-Weinberg expectation that is usually used. Specifically, the *Phi*-statistics analysis we used showed the correlation of some genes or loci within the subdivided lines/populations. In particular, the genetic pattern and structure of the wild population Q (selected within a relatively small area) was similar to the 15 cultivated lines, but was significantly different from the other wild population R (selected across a much larger area). This most likely indicates that these differences in genetic diversity were resulted from bottleneck/drift effects when bringing a small number of individuals into cultivation during breeding practices. Thus, this *Phi*-statistics method could be used further to compare the genotype data of the parent lines against the hybrid lines so as to estimate such bottleneck/drift effects mentioned above. On the other hand, it needs to be noted that when considering wild populations within their natural habitats, sampling methods are one of the major factors affecting the conclusions being drawn about the population genetic structure.

Since blue is the common wild type of floral color for *T*. *fournieri* and *T. conclor*, and our two wild *T. concolor* populations had blue flowers, all the cultivated lines were bred for a specific floral color. In our study, clustering information among cultivated lines and wild populations via our PCoA analysis showed interesting patterns, and much of the variance in genotype among lines is due to differences between certain sets of lines which are most distant from one another. For instance, among cultivated lines I, K, M, N, P and the two wild populations Q and R, there were similar reduction in diversity/heterozygosity from a bottleneck event, and each line/population will retain a similar (but different from each other) content of the wild genetic diversity. As Mehmood et al. (2014) has shown, it is highly probable that the relevant floral color of these lines/populations is associated with a negatively correlated genetic relationship with the wild genetic content mentioned above. Therefore, as the highest loadings in the first two components, strong positive correlations presented in lines A, C, E, M, N, and these cultivated lines have less content of the wild genetic diversity. Consequently, these results from our population genetic analyses could guide future *Torenia* breeding.

Being useful for reconstructing the evolutionary history of lines/populations from population genetic analysis, the population genetic distance can be interpreted as a measure of genetic divergence between species or between lines/populations within a species, whether the distance evaluate the time from common ancestor or the degree of differentiation. Generally, species or wild populations of a species with many similar alleles exhibit small genetic distances due to having had a recent common ancestor. Results from our study indicated that the wild population Q is genetically more closely related to lines M, N, P and they are likely therefore to have had an overall genetic similarity. For the purpose of expanding heterozygosity, these closely related lines/populations should not be used as parent candidates for any inter-line/population crosses, especially those targeting introgressive traits from wild populations. On the contrary, it would be better to cross these closely related lines/populations with other lines/populations containing more genetic differentiation such as A–F.

As previously indicated, this study revealed an extensive genetic diversity and structural pattern within and among the 17 *Torenia* lines/populations studied through the 136 accessions. A large number of specific bands deserve special attention as they were private alleles specific to the line(s)/population(s). The genetic information from these specific alleles indicated significant genetic relationships that separated certain lines/populations, as presented in the above PCoA analysis, phylogenetic dendrogram, and the final population structural bar plot. Furthermore, such population specific polymorphic bands can be excised, then purified from the gel and sequenced to provide more thorough genomic information relevant to the genetic analysis of the population. These specific molecular markers could also be associated with particular phenotypic traits of interest, such as floral color.

Regarding the heterozygosity for 17 *Torenia* populations tested, 15 commercial lines and the wild population Q had low values of Na (number of different alleles), Ne (the number of effective alleles), I (Shannon’s information index), He (expected heterozygosity), and uHe (unbiased expected heterozygosity). Line J has the lowest value of records whilst wild population R had the highest values. Since wild population Q was sampled from a smaller area, and population R was sampled across a much larger area, the different results of these two wild populations could be due to our sampling practices, as suggested above. Furthermore, the mean value of I (0.0758), He (0.0513) and uHe (0.0547) of all the 17 *Torenia* populations tested are considerably less than that reported previously in other genera of plants by Gailite & Rungis (2012), Guo et al. (2014), Baránek et al. (2012), Mehmood et al. (2016), Al-Najm et al. (2016) and Borna et al. (2017). Our lower values indicate significant low genetic diversity/heterozygosity in the 15 *Torenia* series that have been released, and in the wild population Q. This suggests either some kind of bottleneck/drift effect by selection during breeding practices.

Our results also support the view that the retrotransposon-based iPBS system we used could also be used to estimate genetic diversity and structure of other plant species. This would enable population genetic analysis on other biological phenomena such as adaptation, speciation, and population structure. Whilst *Torenia* species have been used previously for frontier research on fertilization and gene engineering of ornamental characteristics within horticultural ecosystems, our research conclusions could complement and expand its use as a model plant for new interests in plant population genetic analysis. Therefore, the results from this study could have two main flow-on effects. The first could be information applicable to selective breeding for commercial purposes. A second application could be with endangered species because the population genetic analysis would be able to index the strength of the population of the species in its natural habitat.

## Conclusions

The iPBS markers showed interesting patterns of genetic variation among wild *T. concolor* populations and *T. fournieri* cultivated lines with different floral color. For cultivated lines and wild populations, there were similar reduction in diversity/heterozygosity from a bottleneck event, and each line/population retained a similar (but different from each other) content of the wild genetic diversity. Variances in genetic diversity/heterozygosity for the two wild populations could be explained by differences in sampling method. Environmental factors could induce transposon activation and generate genetic variability which enabled the acceleration of the evolutionary process of wild *Torenia* species, but selective breeding targeting a specific floral color decreased genetic diversity. As a consequence, this study also provide insight into the effect on genetic variation in a line/population experiencing a bottleneck in which strong phenotypic selection overlaps, but wild *Torenia* populations sampled from broad geographic region represent stronger species strength with outstanding genetic diversity. Using of the iPBS marker system not only revealed genetic variation and population structure for wild and cultivated *Torenia* plants, but this result could guide future selective breeding especially for commercial purposes.

## Supplemental Information

10.7717/peerj.11702/supp-1 Supplemental Information 1 List of 136 *Torenia* accessions from 17 lines/populations used in the iPBS analysis. Note: 15 *T. fournieri* commercial seed lines for population A to P were purchased from Shanghai Yuanyi Seedling Co. Ltd., and two *T. concolor* populations (Q, R) were collected from their natural habitats (Fig. 1).

10.7717/peerj.11702/supp-2 Supplemental Information 2 Dendrogram grouping in relation to floral color of 136 *Torenia* accessions from 17 lines/populations.

10.7717/peerj.11702/supp-3 Supplemental Information 3 First eight components from the PCoA analysis for 17 lines/populations of 136 *Torenia* accessions. In the first three axes or components, high eigen values are marked as bold and the low values are indicated by a subscript. A, Duchess Pink; B, Duchess Burgundy; C, Duchess Deep Blue; D, Kauai Burgundy; E, Kauai Rose; F, Kauai Deep Blue; G, Kauai Blue and White; H, Kauai Magenta; I, Kauai Lemon Drop; J, Kauai White; K, Little Kiss White; L, Little Kiss Burgundy; M, Little Kiss Blue and White; N, Little Kiss Rose Picotee; P, Little Kiss Blue; Q, Lipu and R, Xichou.

10.7717/peerj.11702/supp-4 Supplemental Information 4 Binary matrix of amplification products revealed by seven iPBS primers for 136 accessions of *Torenia* (raw data).

10.7717/peerj.11702/supp-5 Supplemental Information 5 Line/population specific bands in the binary matrix of seven iPBS primers of cultivated Torenia lines (raw data).

10.7717/peerj.11702/supp-6 Supplemental Information 6 Gel images about seven iPBS primers for 136 accessions of *Torenia* (each primer has three images). PCR banding patterns of 136 accessions of Torenia using seven primers.

## Additional Information and Declarations

### Data Availability

The following information was supplied regarding data availability: The raw measurements and raw data are available in the Supplemental Files.
