# Supplementary material for: Genetic analysis and population structure of wild and cultivated wishbone flower (Torenia fournieri Lind.) lines related to specific floral color
Source: PeerJ. 2021 Jul 5;9:e11702. doi: 10.7717/peerj.11702 (PMC8265383; doi:10.7717/peerj.11702)
Supplement: Supplemental Information 1 — Note: 15 T. fournieri commercial seed lines for population A to P were purchased from Shanghai Yuanyi Seedling Co. Ltd., and two T. concolor populations (Q, R) were collected from their natural habitats (Fig. 1). [file peerj-09-11702-s001.docx]

**Supplemental File 1:**

**List of 136 *Torenia* accessions from 17 lines/populations used in the iPBS analysis.**

| Accession ID | Population ID | Origin | Genus | Species | Floral color |
| --- | --- | --- | --- | --- | --- |
| A1 | 1 | Duchess Pink | *T.* | *fournieri*Lind | Pink |
| A2 | 1 | Duchess Pink | *T.* | *fournieri* | Pink |
| A3 | 1 | Duchess Pink | *T.* | *fournieri* | Pink |
| A4 | 1 | Duchess Pink | *T.* | *fournieri* | Pink |
| A5 | 1 | Duchess Pink | *T.* | *fournieri* | Pink |
| A6 | 1 | Duchess Pink | *T.* | *fournieri* | Pink |
| A7 | 1 | Duchess Pink | *T.* | *fournieri* | Pink |
| A8 | 1 | Duchess Pink | *T.* | *fournieri* | Pink |
| B9 | 2 | Duchess Burgundy | *T.* | *fournieri* | Burgundy |
| B10 | 2 | Duchess Burgundy | *T.* | *fournieri* | Burgundy |
| B11 | 2 | Duchess Burgundy | *T.* | *fournieri* | Burgundy |
| B12 | 2 | Duchess Burgundy | *T.* | *fournieri* | Burgundy |
| B13 | 2 | Duchess Burgundy | *T.* | *fournieri* | Burgundy |
| B14 | 2 | Duchess Burgundy | *T.* | *fournieri* | Burgundy |
| B15 | 2 | Duchess Burgundy | *T.* | *fournieri* | Burgundy |
| B16 | 2 | Duchess Burgundy | *T.* | *fournieri* | Burgundy |
| C17 | 3 | Duchess Deep Blue | *T.* | *fournieri* | Deep Blue |
| C18 | 3 | Duchess Deep Blue | *T.* | *fournieri* | Deep Blue |
| C19 | 3 | Duchess Deep Blue | *T.* | *fournieri* | Deep Blue |
| C20 | 3 | Duchess Deep Blue | *T.* | *fournieri* | Deep Blue |
| C21 | 3 | Duchess Deep Blue | *T.* | *fournieri* | Deep Blue |
| C22 | 3 | Duchess Deep Blue | *T.* | *fournieri* | Deep Blue |
| C23 | 3 | Duchess Deep Blue | *T.* | *fournieri* | Deep Blue |
| C24 | 3 | Duchess Deep Blue | *T.* | *fournieri* | Deep Blue |
| D25 | 4 | Kauai Burgundy | *T.* | *fournieri* | Burgundy |
| D26 | 4 | Kauai Burgundy | *T.* | *fournieri* | Burgundy |
| D27 | 4 | Kauai Burgundy | *T.* | *fournieri* | Burgundy |
| D28 | 4 | Kauai Burgundy | *T.* | *fournieri* | Burgundy |
| D29 | 4 | Kauai Burgundy | *T.* | *fournieri* | Burgundy |
| D30 | 4 | Kauai Burgundy | *T.* | *fournieri* | Burgundy |
| D31 | 4 | Kauai Burgundy | *T.* | *fournieri* | Burgundy |
| D32 | 4 | Kauai Burgundy | *T.* | *fournieri* | Burgundy |
| E33 | 5 | Kauai Rose | *T.* | *fournieri* | Rose |
| E34 | 5 | Kauai Rose | *T.* | *fournieri* | Rose |
| E35 | 5 | Kauai Rose | *T.* | *fournieri* | Rose |
| E36 | 5 | Kauai Rose | *T.* | *fournieri* | Rose |
| E37 | 5 | Kauai Rose | *T.* | *fournieri* | Rose |
| E38 | 5 | Kauai Rose | *T.* | *fournieri* | Rose |
| E39 | 5 | Kauai Rose | *T.* | *fournieri* | Rose |
| E40 | 5 | Kauai Rose | *T.* | *fournieri* | Rose |
| F41 | 6 | Kauai Deep Blue | *T.* | *fournieri* | Deep Blue |
| F42 | 6 | Kauai Deep Blue | *T.* | *fournieri* | Deep Blue |
| F43 | 6 | Kauai Deep Blue | *T.* | *fournieri* | Deep Blue |
| F44 | 6 | Kauai Deep Blue | *T.* | *fournieri* | Deep Blue |
| F45 | 6 | Kauai Deep Blue | *T.* | *fournieri* | Deep Blue |
| F46 | 6 | Kauai Deep Blue | *T.* | *fournieri* | Deep Blue |
| F47 | 6 | Kauai Deep Blue | *T.* | *fournieri* | Deep Blue |
| F48 | 6 | Kauai Deep Blue | *T.* | *fournieri* | Deep Blue |
| G49 | 7 | Kauai Blue and White | *T.* | *fournieri* | Blue and White |
| G50 | 7 | Kauai Blue and White | *T.* | *fournieri* | Blue and White |
| G51 | 7 | Kauai Blue and White | *T.* | *fournieri* | Blue and White |
| G52 | 7 | Kauai Blue and White | *T.* | *fournieri* | Blue and White |
| G53 | 7 | Kauai Blue and White | *T.* | *fournieri* | Blue and White |
| G54 | 7 | Kauai Blue and White | *T.* | *fournieri* | Blue and White |
| G55 | 7 | Kauai Blue and White | *T.* | *fournieri* | Blue and White |
| G56 | 7 | Kauai Blue and White | *T.* | *fournieri* | Blue and White |
| H57 | 8 | Kauai Magenta | *T.* | *fournieri* | Magenta |
| H58 | 8 | Kauai Magenta | *T.* | *fournieri* | Magenta |
| H59 | 8 | Kauai Magenta | *T.* | *fournieri* | Magenta |
| H60 | 8 | Kauai Magenta | *T.* | *fournieri* | Magenta |
| H61 | 8 | Kauai Magenta | *T.* | *fournieri* | Magenta |
| H62 | 8 | Kauai Magenta | *T.* | *fournieri* | Magenta |
| H63 | 8 | Kauai Magenta | *T.* | *fournieri* | Magenta |
| H64 | 8 | Kauai Magenta | *T.* | *fournieri* | Magenta |
| I65 | 9 | Kauai Lemon Drop | *T.* | *fournieri* | Lemon |
| I66 | 9 | Kauai Lemon Drop | *T.* | *fournieri* | Lemon |
| I67 | 9 | Kauai Lemon Drop | *T.* | *fournieri* | Lemon |
| I68 | 9 | Kauai Lemon Drop | *T.* | *fournieri* | Lemon |
| I69 | 9 | Kauai Lemon Drop | *T.* | *fournieri* | Lemon |
| I70 | 9 | Kauai Lemon Drop | *T.* | *fournieri* | Lemon |
| I71 | 9 | Kauai Lemon Drop | *T.* | *fournieri* | Lemon |
| I72 | 9 | Kauai Lemon Drop | *T.* | *fournieri* | Lemon |
| J73 | 10 | Kauai White | *T.* | *fournieri* | White |
| J74 | 10 | Kauai White | *T.* | *fournieri* | White |
| J75 | 10 | Kauai White | *T.* | *fournieri* | White |
| J76 | 10 | Kauai White | *T.* | *fournieri* | White |
| J77 | 10 | Kauai White | *T.* | *fournieri* | White |
| J78 | 10 | Kauai White | *T.* | *fournieri* | White |
| J79 | 10 | Kauai White | *T.* | *fournieri* | White |
| J80 | 10 | Kauai White | *T.* | *fournieri* | White |
| K81 | 11 | Little Kiss White | *T.* | *fournieri* | White |
| K82 | 11 | Little Kiss White | *T.* | *fournieri* | White |
| K83 | 11 | Little Kiss White | *T.* | *fournieri* | White |
| K84 | 11 | Little Kiss White | *T.* | *fournieri* | White |
| K85 | 11 | Little Kiss White | *T.* | *fournieri* | White |
| K86 | 11 | Little Kiss White | *T.* | *fournieri* | White |
| K87 | 11 | Little Kiss White | *T.* | *fournieri* | White |
| K88 | 11 | Little Kiss White | *T.* | *fournieri* | White |
| L89 | 12 | Little Kiss Burgundy | *T.* | *fournieri* | Burgundy |
| L90 | 12 | Little Kiss Burgundy | *T.* | *fournieri* | Burgundy |
| L91 | 12 | Little Kiss Burgundy | *T.* | *fournieri* | Burgundy |
| L92 | 12 | Little Kiss Burgundy | *T.* | *fournieri* | Burgundy |
| L93 | 12 | Little Kiss Burgundy | *T.* | *fournieri* | Burgundy |
| L94 | 12 | Little Kiss Burgundy | *T.* | *fournieri* | Burgundy |
| L95 | 12 | Little Kiss Burgundy | *T.* | *fournieri* | Burgundy |
| L96 | 12 | Little Kiss Burgundy | *T.* | *fournieri* | Burgundy |
| M97 | 13 | Little Kiss Blue and White | *T.* | *fournieri* | Blue and White |
| M98 | 13 | Little Kiss Blue and White | *T.* | *fournieri* | Blue and White |
| M99 | 13 | Little Kiss Blue and White | *T.* | *fournieri* | Blue and White |
| M100 | 13 | Little Kiss Blue and White | *T.* | *fournieri* | Blue and White |
| M101 | 13 | Little Kiss Blue and White | *T.* | *fournieri* | Blue and White |
| M102 | 13 | Little Kiss Blue and White | *T.* | *fournieri* | Blue and White |
| M103 | 13 | Little Kiss Blue and White | *T.* | *fournieri* | Blue and White |
| M104 | 13 | Little Kiss Blue and White | *T.* | *fournieri* | Blue and White |
| N105 | 14 | Little Kiss Rose Picotee | *T.* | *fournieri* | Rose Picotee |
| N106 | 14 | Little Kiss Rose Picotee | *T.* | *fournieri* | Rose Picotee |
| N107 | 14 | Little Kiss Rose Picotee | *T.* | *fournieri* | Rose Picotee |
| N108 | 14 | Little Kiss Rose Picotee | *T.* | *fournieri* | Rose Picotee |
| N109 | 14 | Little Kiss Rose Picotee | *T.* | *fournieri* | Rose Picotee |
| N110 | 14 | Little Kiss Rose Picotee | *T.* | *fournieri* | Rose Picotee |
| N111 | 14 | Little Kiss Rose Picotee | *T.* | *fournieri* | Rose Picotee |
| N112 | 14 | Little Kiss Rose Picotee | *T.* | *fournieri* | Rose Picotee |
| P113 | 15 | Little Kiss Blue | *T.* | *fournieri* | Blue |
| P114 | 15 | Little Kiss Blue | *T.* | *fournieri* | Blue |
| P115 | 15 | Little Kiss Blue | *T.* | *fournieri* | Blue |
| P116 | 15 | Little Kiss Blue | *T.* | *fournieri* | Blue |
| P117 | 15 | Little Kiss Blue | *T.* | *fournieri* | Blue |
| P118 | 15 | Little Kiss Blue | *T.* | *fournieri* | Blue |
| P119 | 15 | Little Kiss Blue | *T.* | *fournieri* | Blue |
| P120 | 15 | Little Kiss Blue | *T.* | *fournieri* | Blue |
| Q121 | 16 | Lipu, Guangxi | *T.* | *concolor* Lindl. | Blue |
| Q122 | 16 | Lipu, Guangxi | *T.* | *concolor* | Blue |
| Q123 | 16 | Lipu, Guangxi | *T.* | *concolor* | Blue |
| Q124 | 16 | Lipu, Guangxi | *T.* | *concolor* | Blue |
| Q125 | 16 | Lipu, Guangxi | *T.* | *concolor* | Blue |
| Q126 | 16 | Lipu, Guangxi | *T.* | *concolor* | Blue |
| Q127 | 16 | Lipu, Guangxi | *T.* | *concolor* | Blue |
| Q128 | 16 | Lipu, Guangxi | *T.* | *concolor* | Blue |
| R129 | 17 | Xichou, Yunan | *T.* | *concolor* | Blue |
| R130 | 17 | Xichou, Yunan | *T.* | *concolor* | Blue |
| R131 | 17 | Xichou, Yunan | *T.* | *concolor* | Blue |
| R132 | 17 | Xichou, Yunan | *T.* | *concolor* | Blue |
| R133 | 17 | Xichou, Yunan | *T.* | *concolor* | Blue |
| R134 | 17 | Xichou, Yunan | *T.* | *concolor* | Blue |
| R135 | 17 | Xichou, Yunan | *T.* | *concolor* | Blue |
| R136 | 17 | Xichou, Yunan | *T.* | *concolor* | Blue |

Note: 15 *T. fournieri* commercial seed lines for population A to P were purchased from Shanghai Yuanyi Seedling Co. Ltd., and 2 *T. concolor* populations (Q, R) were collected from their natural habitats (Figure 1).
