# Supplementary material for: Genetic analysis and population structure of wild and cultivated wishbone flower (Torenia fournieri Lind.) lines related to specific floral color
Source: PeerJ. 2021 Jul 5;9:e11702. doi: 10.7717/peerj.11702 (PMC8265383; doi:10.7717/peerj.11702)
Supplement: Supplemental Information 2 [file peerj-09-11702-s002.docx]

**Supplemental File 2:**

**Dendrogram grouping** **in relation to floral color of 136 *Torenia* accessions from 17 lines/populations.**

| Clusters | Genotypes | Floral color of populations |
| --- | --- | --- |
| Group 1 | C4, C7, C3, C5, C6, C1, C2, C8; E2, E7, E1, E3, E5, E4, E6, E8; A5, A1, A2, A3, A8, A7, A4, A6.  F8, F1, F7, F2, F3, F5, F4, F6; D6, D7, D8, D1, D2, D3, D4, D5; B2, B3, B1, B8, B7, B4, B5, B6. | Duchess Deep Blue, Kauai Rose, Duchess Pink.  Kauai Deep Blue, Kauai Burgundy, Duchess Burgundy. |
| Group 2 | H4, H5, H7, H3, H8, H2, H1, H6; L5, L6, L4, L7, L8, L1, L2, L3; G1, G2, G4, G5, G8, G6, G7, G3.  K6, K2, K1, K7, K5, K3, K8, K4; I8, I7, I6, I5, I1, I3, I4, I2; J8, J6, J4, J7, J5, J3, J2, J1. | Kauai Magenta, Little Kiss Burgundy, Kauai Blue and White.  Little Kiss White, Kauai lemon Drop, Kauai White. |
| Group 3 | N8, N7, N5, N4, N6, N1, N3, N2; M8, M7, M6, M4, M5, M1, M3, M2; P7, P6, P4, P5, P8, P3, P2, P1. | Litle Kiss: Rose Picotee, Blue and White, Blue. |
| Group 4 | Q4, Q3, Q2, Q1, Q6, Q8, Q7, Q5. | Wild: Lipu Deep Blue |
| Group 5 | R5, R4, R7, R6, R3, R2, R1, R8. | Wild: Xichou Deep Blue |
