# Supplementary material for: Genetic analysis and population structure of wild and cultivated wishbone flower (Torenia fournieri Lind.) lines related to specific floral color
Source: PeerJ. 2021 Jul 5;9:e11702. doi: 10.7717/peerj.11702 (PMC8265383; doi:10.7717/peerj.11702)
Supplement: Supplemental Information 3 — In the first three axes or components, high eigen values are marked as bold and the low values are indicated by a subscript. A, Duchess Pink; B, Duchess Burgundy; C, Duchess Deep Blue; D, Kauai Burgundy; E, Kauai Rose; F, Kauai Deep Blue; G, Kauai Blue and White; H, Kauai Magenta; I, Kauai Lemon Drop; J, Kauai White; K, Little Kiss White; L, Little Kiss Burgundy; M, Little Kiss Blue and White; N, Little Kiss Rose Picotee; P, Little Kiss Blue; Q, Lipu and R, Xichou. [file peerj-09-11702-s003.docx]

**Supplemental File 3:**

**First eight components from the PCoA analysis for 17 lines/populations of 136 *Torenia* accessions.**

| Populations | 1 | 2 | 3 | 4 | 5 | 6 | 7 | 8 |
| --- | --- | --- | --- | --- | --- | --- | --- | --- |
| A1 | **0.286** | 0.142 | -0.099 | 0.076 | -0.056 | -0.274 | -0.174 | 0.157 |
| A2 | **0.436** | 0.220 | 0.051 | 0.059 | -0.131 | -0.183 | -0.057 | 0.184 |
| A3 | **0.487** | 0.208 | 0.046 | 0.052 | -0.108 | -0.170 | -0.067 | 0.211 |
| A4 | **0.607** | 0.274 | 0.256 | -0.012 | -0.092 | -0.001 | 0.022 | 0.314 |
| A5 | **0.530** | 0.123 | 0.343 | 0.027 | -0.078 | -0.041 | 0.106 | 0.207 |
| A6 | **0.629** | 0.245 | 0.187 | 0.015 | -0.170 | -0.059 | 0.061 | 0.250 |
| A7 | **0.612** | 0.246 | 0.254 | 0.011 | -0.076 | -0.130 | -0.070 | 0.290 |
| A8 | **0.469** | 0.273 | 0.161 | 0.027 | -0.035 | -0.023 | -0.073 | 0.298 |
| B1 | 0.326 | 0.194 | 0.245 | 0.274 | 0.744 | 0.010 | 0.411 | 0.056 |
| B2 | 0.273 | 0.133 | 0.230 | 0.282 | 0.755 | 0.021 | 0.535 | 0.257 |
| B3 | 0.294 | 0.165 | 0.273 | 0.253 | 0.777 | 0.130 | 0.547 | 0.013 |
| B4 | 0.444 | 0.157 | 0.291 | 0.233 | 0.724 | 0.075 | 0.613 | 0.222 |
| B5 | 0.369 | 0.158 | 0.331 | 0.310 | 0.783 | 0.128 | 0.616 | 0.170 |
| B6 | 0.369 | 0.158 | 0.331 | 0.310 | 0.783 | 0.128 | 0.616 | 0.170 |
| B7 | 0.477 | 0.154 | 0.277 | 0.349 | 0.702 | 0.021 | 0.482 | 0.147 |
| B8 | 0.366 | 0.124 | 0.277 | 0.283 | 0.760 | 0.072 | 0.495 | 0.001 |
| C1 | **0.491** | 0.306 | 0.281 | 0.031 | -0.395 | 0.116 | 0.047 | 0.112 |
| C2 | **0.509** | 0.276 | 0.252 | 0.024 | -0.408 | 0.120 | -0.002 | 0.091 |
| C3 | **0.615** | 0.280 | 0.292 | -0.045 | -0.421 | 0.213 | 0.082 | 0.100 |
| C4 | **0.634** | 0.304 | 0.319 | -0.041 | -0.448 | 0.215 | 0.051 | 0.114 |
| C5 | **0.620** | 0.316 | 0.328 | 0.016 | -0.433 | 0.179 | 0.076 | 0.029 |
| C6 | **0.664** | 0.319 | 0.339 | -0.039 | -0.438 | 0.199 | 0.044 | 0.034 |
| C7 | **0.605** | 0.310 | 0.318 | 0.012 | -0.452 | 0.216 | 0.057 | 0.059 |
| C8 | **0.617** | 0.356 | 0.339 | 0.060 | -0.356 | 0.211 | 0.144 | 0.007 |
| D1 | 0.478 | 0.402 | **0.655** | 0.284 | 0.141 | 0.162 | -0.201 | 0.005 |
| D2 | 0.501 | 0.423 | **0.631** | 0.211 | 0.029 | 0.246 | -0.282 | 0.026 |
| D3 | 0.478 | 0.383 | **0.637** | 0.178 | 0.097 | 0.244 | -0.320 | 0.082 |
| D4 | 0.489 | 0.385 | **0.641** | 0.182 | 0.141 | 0.267 | -0.279 | 0.045 |
| D5 | 0.442 | 0.324 | **0.578** | 0.153 | 0.166 | 0.275 | -0.292 | 0.033 |
| D6 | 0.434 | 0.365 | **0.466** | 0.221 | 0.083 | 0.192 | -0.346 | -0.093 |
| D7 | 0.398 | 0.346 | **0.483** | 0.217 | 0.196 | 0.234 | -0.395 | -0.040 |
| D8 | 0.394 | 0.371 | **0.493** | 0.157 | 0.186 | 0.273 | -0.404 | 0.096 |
| E1 | **0.540** | **0.438** | 0.368 | -0.075 | -0.230 | -0.271 | -0.344 | 0.096 |
| E2 | **0.527** | **0.457** | 0.366 | -0.016 | -0.295 | -0.115 | -0.191 | 0.027 |
| E3 | **0.518** | **0.369** | 0.237 | 0.009 | -0.285 | -0.326 | -0.262 | 0.073 |
| E4 | **0.630** | **0.425** | 0.414 | -0.012 | -0.244 | -0.137 | -0.075 | 0.134 |
| E5 | **0.644** | **0.517** | 0.438 | 0.033 | -0.266 | -0.014 | -0.076 | 0.100 |
| E6 | **0.532** | **0.451** | 0.337 | 0.040 | -0.207 | -0.183 | -0.251 | -0.012 |
| E7 | **0.581** | **0.507** | 0.471 | -0.021 | -0.287 | -0.128 | -0.198 | 0.015 |
| E8 | **0.566** | **0.434** | 0.303 | 0.046 | -0.235 | -0.222 | -0.203 | 0.071 |
| F1 | 0.481 | 0.270 | 0.365 | 0.075 | -0.246 | -0.221 | 0.130 | -0.330 |
| F2 | 0.511 | 0.339 | 0.356 | -0.070 | -0.257 | -0.290 | 0.138 | -0.282 |
| F3 | 0.500 | 0.217 | 0.379 | 0.095 | -0.052 | -0.255 | 0.216 | -0.442 |
| F4 | 0.532 | 0.220 | 0.458 | -0.004 | -0.129 | -0.255 | 0.041 | -0.435 |
| F5 | 0.513 | 0.182 | 0.396 | 0.086 | 0.000 | -0.343 | 0.157 | -0.522 |
| F6 | 0.449 | 0.228 | 0.395 | 0.066 | -0.094 | -0.276 | 0.131 | -0.661 |
| F7 | 0.391 | 0.254 | 0.258 | -0.022 | -0.228 | -0.216 | -0.081 | -0.571 |
| F8 | 0.391 | 0.078 | 0.477 | -0.054 | -0.210 | -0.285 | -0.023 | -0.352 |
| G1 | 0.183 | -0.317 | -0.459 | -0.254 | 0.051 | 0.278 | -0.164 | -0.602 |
| G2 | 0.217 | -0.282 | -0.435 | -0.303 | 0.021 | 0.260 | -0.021 | -0.621 |
| G3 | 0.148 | -0.362 | -0.300 | -0.228 | 0.163 | 0.234 | -0.009 | -0.542 |
| G4 | 0.146 | -0.285 | -0.428 | -0.250 | 0.093 | 0.247 | -0.034 | -0.688 |
| G5 | 0.254 | -0.325 | -0.354 | -0.252 | 0.118 | 0.218 | 0.064 | -0.632 |
| G6 | 0.327 | -0.271 | -0.297 | -0.183 | 0.094 | 0.258 | -0.040 | -0.653 |
| G7 | 0.333 | -0.249 | -0.336 | -0.176 | -0.010 | 0.188 | 0.043 | -0.688 |
| G8 | 0.478 | -0.414 | -0.343 | -0.204 | 0.032 | 0.219 | 0.040 | -0.570 |
| H1 | 0.267 | -0.318 | -0.262 | 0.086 | 0.477 | 0.247 | -0.423 | 0.037 |
| H2 | 0.272 | -0.311 | -0.303 | 0.044 | 0.368 | 0.305 | -0.415 | 0.102 |
| H3 | 0.288 | -0.314 | -0.302 | 0.097 | 0.318 | 0.390 | -0.490 | 0.092 |
| H4 | 0.210 | -0.254 | -0.240 | 0.120 | 0.518 | 0.413 | -0.578 | 0.176 |
| H5 | 0.250 | -0.262 | -0.232 | 0.110 | 0.507 | 0.468 | -0.536 | 0.123 |
| H6 | -0.027 | -0.380 | -0.346 | 0.066 | 0.498 | 0.379 | -0.499 | -0.007 |
| H7 | 0.226 | -0.324 | -0.253 | 0.068 | 0.522 | 0.365 | -0.602 | 0.141 |
| H8 | 0.138 | -0.336 | -0.326 | 0.034 | 0.353 | 0.401 | -0.511 | -0.016 |
| I1 | 0.306 | -0.622 | -0.379 | -0.283 | -0.172 | 0.396 | 0.080 | 0.183 |
| I2 | 0.377 | -0.628 | -0.314 | -0.347 | -0.231 | 0.400 | 0.193 | 0.095 |
| I3 | 0.388 | -0.603 | -0.292 | -0.366 | -0.180 | 0.413 | 0.167 | 0.085 |
| I4 | 0.377 | -0.628 | -0.314 | -0.347 | -0.231 | 0.400 | 0.193 | 0.095 |
| I5 | 0.401 | -0.638 | -0.361 | -0.324 | -0.296 | 0.359 | 0.167 | 0.141 |
| I6 | 0.434 | -0.639 | -0.317 | -0.359 | -0.277 | 0.350 | 0.179 | 0.119 |
| I7 | 0.449 | -0.638 | -0.357 | -0.329 | -0.308 | 0.344 | 0.205 | 0.150 |
| I8 | 0.482 | -0.639 | -0.313 | -0.363 | -0.289 | 0.336 | 0.218 | 0.128 |
| J1 | 0.373 | -0.292 | -0.307 | -0.149 | -0.123 | -0.087 | 0.394 | 0.172 |
| J2 | 0.373 | -0.292 | -0.307 | -0.149 | -0.123 | -0.087 | 0.394 | 0.172 |
| J3 | 0.353 | -0.303 | -0.331 | -0.147 | -0.116 | -0.149 | 0.323 | 0.109 |
| J4 | 0.445 | -0.279 | -0.277 | -0.154 | -0.111 | -0.053 | 0.367 | 0.086 |
| J5 | 0.357 | -0.284 | -0.343 | -0.118 | -0.142 | -0.102 | 0.358 | 0.138 |
| J6 | 0.445 | -0.279 | -0.277 | -0.154 | -0.111 | -0.053 | 0.367 | 0.086 |
| J7 | 0.394 | -0.267 | -0.272 | -0.147 | -0.134 | -0.066 | 0.378 | 0.058 |
| J8 | 0.445 | -0.279 | -0.277 | -0.154 | -0.111 | -0.053 | 0.367 | 0.086 |
| K1 | 0.189 | -0.476 | -0.557 | -0.121 | -0.274 | -0.309 | -0.097 | 0.159 |
| K2 | 0.194 | -0.434 | -0.548 | -0.098 | -0.151 | -0.274 | -0.139 | 0.203 |
| K3 | 0.206 | -0.464 | -0.598 | -0.072 | -0.126 | -0.186 | -0.093 | 0.299 |
| K4 | 0.207 | -0.538 | -0.586 | -0.165 | -0.175 | -0.280 | -0.096 | 0.300 |
| K5 | 0.329 | -0.496 | -0.534 | -0.158 | -0.115 | -0.224 | -0.120 | 0.283 |
| K6 | 0.281 | -0.523 | -0.508 | -0.236 | -0.159 | -0.317 | -0.175 | 0.229 |
| K7 | 0.338 | -0.499 | -0.558 | -0.133 | -0.148 | -0.223 | -0.061 | 0.244 |
| K8 | 0.271 | -0.535 | -0.588 | -0.142 | -0.196 | -0.222 | -0.040 | 0.232 |
| L1 | 0.168 | -0.203 | -0.335 | 0.011 | 0.374 | -0.515 | -0.267 | 0.011 |
| L2 | 0.100 | -0.206 | -0.398 | 0.064 | 0.390 | -0.603 | -0.282 | 0.091 |
| L3 | 0.179 | -0.227 | -0.379 | 0.020 | 0.253 | -0.592 | -0.213 | -0.007 |
| L4 | 0.152 | -0.191 | -0.302 | -0.028 | 0.342 | -0.786 | -0.136 | -0.060 |
| L5 | 0.160 | -0.226 | -0.330 | -0.044 | 0.331 | -0.800 | -0.139 | -0.035 |
| L6 | 0.138 | -0.235 | -0.349 | 0.004 | 0.358 | -0.770 | -0.173 | -0.131 |
| L7 | 0.120 | -0.244 | -0.305 | 0.012 | 0.333 | -0.711 | -0.149 | -0.109 |
| L8 | 0.190 | -0.160 | -0.246 | 0.019 | 0.335 | -0.691 | -0.025 | -0.182 |
| M1 | -0.800 | **0.465** | -0.409 | 0.241 | -0.010 | -0.126 | 0.369 | -0.078 |
| M2 | -0.743 | **0.478** | -0.387 | 0.273 | -0.093 | 0.050 | 0.280 | -0.008 |
| M3 | -0.802 | **0.484** | -0.506 | 0.431 | -0.133 | 0.024 | 0.329 | 0.002 |
| M4 | -0.795 | **0.478** | -0.355 | 0.385 | -0.111 | 0.117 | 0.187 | -0.176 |
| M5 | -0.832 | **0.557** | -0.440 | 0.401 | -0.184 | 0.033 | 0.155 | -0.072 |
| M6 | -0.816 | **0.488** | -0.411 | 0.377 | -0.156 | 0.080 | 0.024 | -0.191 |
| M7 | -0.826 | **0.489** | -0.376 | 0.360 | -0.032 | 0.009 | 0.189 | -0.113 |
| M8 | -0.854 | **0.533** | -0.355 | 0.399 | 0.100 | 0.124 | 0.054 | -0.122 |
| N1 | -0.788 | **0.500** | -0.414 | 0.192 | -0.105 | -0.037 | -0.179 | 0.068 |
| N2 | -0.810 | **0.541** | -0.425 | 0.219 | -0.019 | -0.094 | -0.191 | 0.066 |
| N3 | -0.874 | **0.525** | -0.425 | 0.213 | 0.022 | -0.030 | -0.106 | 0.085 |
| N4 | -0.836 | **0.492** | -0.404 | 0.285 | 0.022 | -0.003 | -0.220 | 0.090 |
| N5 | -0.876 | **0.509** | -0.406 | 0.299 | 0.011 | -0.045 | -0.198 | 0.101 |
| N6 | -0.765 | **0.472** | -0.363 | 0.271 | -0.159 | 0.001 | -0.279 | 0.095 |
| N7 | -0.894 | **0.506** | -0.370 | 0.304 | -0.155 | 0.049 | -0.246 | 0.089 |
| N8 | -0.825 | **0.519** | -0.406 | 0.232 | -0.060 | -0.069 | -0.163 | 0.107 |
| P1 | -0.724 | 0.467 | -0.437 | 0.310 | -0.216 | -0.008 | 0.226 | 0.095 |
| P2 | -0.740 | 0.251 | -0.480 | 0.277 | -0.117 | -0.033 | 0.155 | 0.045 |
| P3 | -0.841 | 0.364 | -0.460 | 0.351 | -0.072 | 0.137 | 0.157 | 0.092 |
| P4 | -0.787 | 0.318 | -0.437 | 0.379 | -0.186 | 0.195 | 0.040 | -0.100 |
| P5 | -0.734 | 0.403 | -0.449 | 0.430 | -0.152 | 0.203 | 0.062 | -0.045 |
| P6 | -0.839 | 0.366 | -0.393 | 0.428 | -0.196 | 0.299 | 0.045 | -0.030 |
| P7 | -0.895 | 0.387 | -0.457 | 0.381 | -0.066 | 0.232 | 0.111 | -0.045 |
| P8 | -0.880 | 0.429 | -0.472 | 0.432 | -0.213 | 0.183 | 0.078 | -0.048 |
| Q1 | -1.116 | 0.284 | **0.404** | -1.177 | 0.121 | -0.011 | 0.057 | 0.118 |
| Q2 | -1.145 | 0.279 | **0.452** | -1.304 | 0.188 | -0.019 | 0.020 | 0.039 |
| Q3 | -1.061 | 0.279 | **0.495** | -1.342 | 0.207 | 0.012 | 0.056 | 0.040 |
| Q4 | -1.118 | 0.294 | **0.462** | -1.300 | 0.239 | -0.012 | 0.043 | 0.055 |
| Q5 | -1.144 | 0.144 | **0.499** | -1.332 | 0.073 | -0.078 | 0.030 | -0.022 |
| Q6 | -1.054 | 0.207 | **0.382** | -1.236 | 0.175 | 0.024 | -0.090 | -0.039 |
| Q7 | -1.086 | 0.281 | **0.466** | -1.247 | 0.127 | 0.036 | -0.032 | 0.144 |
| Q8 | -1.092 | 0.359 | **0.516** | -1.272 | 0.129 | 0.031 | -0.015 | 0.161 |
| R1 | -1.008 | -1.182 | **1.007** | 0.528 | -0.168 | -0.046 | -0.138 | -0.057 |
| R2 | -1.037 | -1.184 | **0.916** | 0.685 | -0.245 | -0.074 | 0.070 | 0.112 |
| R3 | -1.070 | -1.230 | **0.918** | 0.769 | -0.217 | -0.027 | 0.124 | 0.083 |
| R4 | -1.101 | -1.206 | **1.000** | 0.645 | -0.148 | -0.018 | 0.096 | -0.056 |
| R5 | -1.035 | -1.194 | **0.988** | 0.529 | -0.173 | -0.006 | -0.019 | -0.042 |
| R6 | -1.022 | -1.006 | **0.952** | 0.439 | 0.010 | -0.046 | -0.176 | -0.008 |
| R7 | -1.077 | -1.219 | **0.977** | 0.520 | -0.121 | -0.097 | -0.143 | -0.064 |
| R8 | -1.237 | -0.781 | **0.456** | 0.066 | -0.158 | -0.204 | 0.117 | -0.009 |
|  |  |  |  |  |  |  |  |  |
| Variability (%) | 51.486 | 28.401 | 26.197 | 21.123 | 11.313 | 9.410 | 8.674 | 6.883 |

In the first three axes or components, high eigen values are marked as bold and the low values are indicated by a subscript. A: Duchess Pink; B: Duchess Burgundy; C: Duchess Deep Blue; D: Kauai Burgundy; E: Kauai Rose; F: Kauai Deep Blue; G: Kauai Blue and White; H: Kauai Magenta; I: Kauai Lemon Drop; J: Kauai White; K: Little Kiss White; L: Little Kiss Burgundy; M: Little Kiss Blue and White; N: Little Kiss Rose Picotee; P: Little Kiss Blue; Q: Lipu and R: Xichou.
