# Supplementary figures and images for: Genetic analysis and population structure of wild and cultivated wishbone flower (Torenia fournieri Lind.) lines related to specific floral color
Source: PeerJ. 2021 Jul 5;9:e11702. doi: 10.7717/peerj.11702 (PMC8265383; doi:10.7717/peerj.11702)

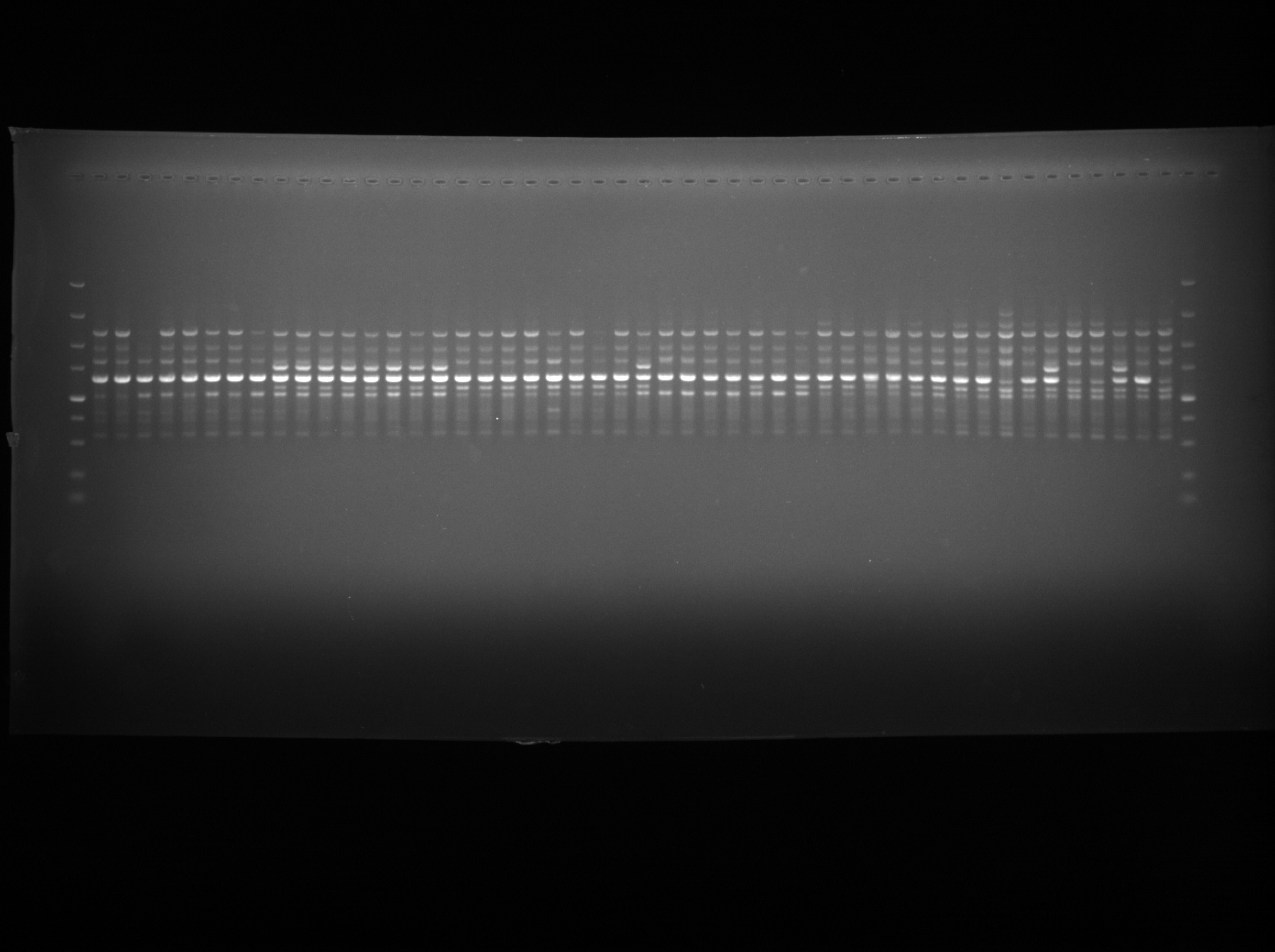

Supplement: Supplemental Information 6 — PCR banding patterns of 136 accessions of Torenia using seven primers. [file peerj-09-11702-s006.zip › iPBS_electrophoresis/Primer 2076 accessions 1-48.jpg]

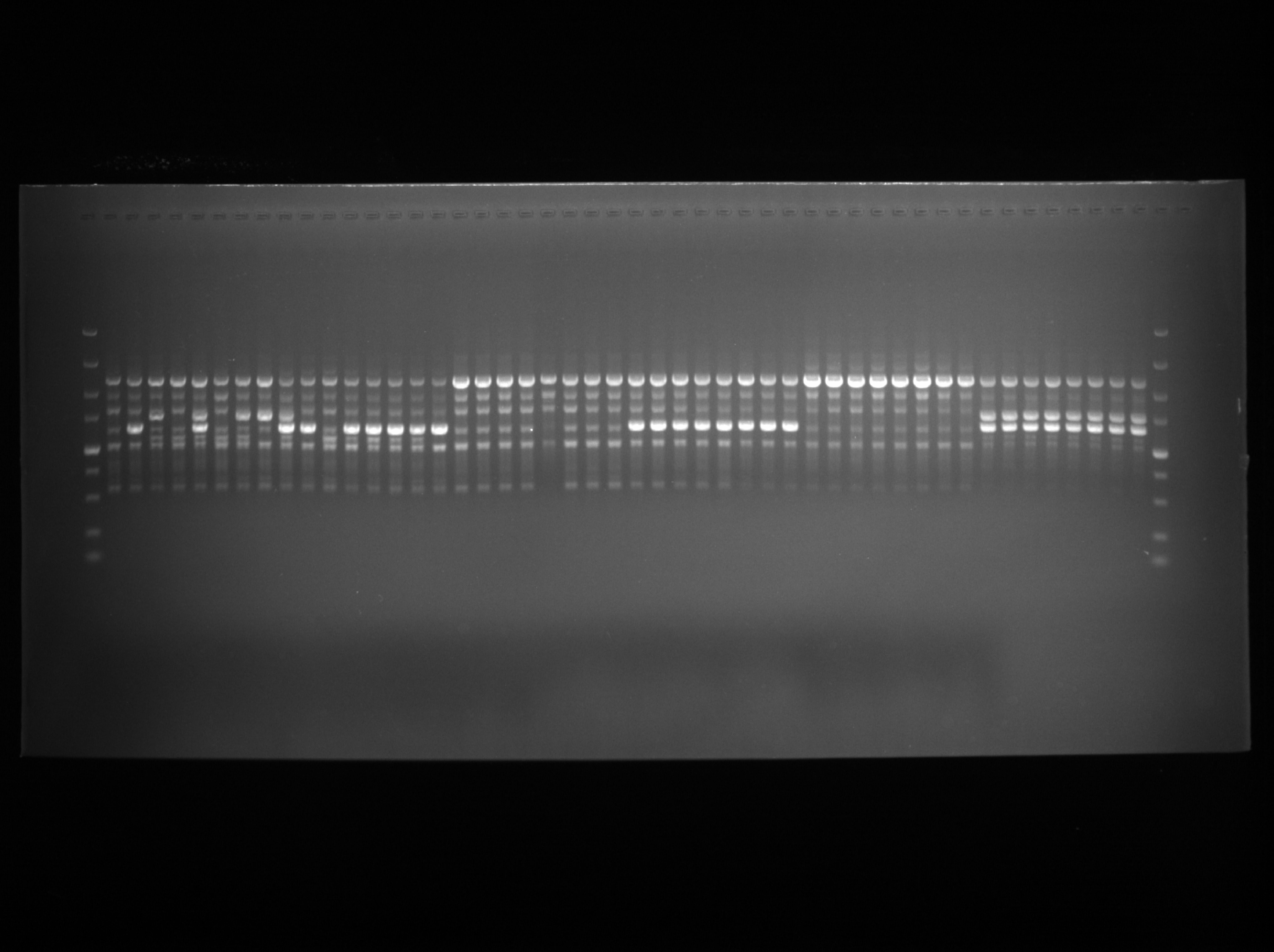

Supplement: Supplemental Information 6 — PCR banding patterns of 136 accessions of Torenia using seven primers. [file peerj-09-11702-s006.zip › iPBS_electrophoresis/Primer 2076 accessions 49-96.jpg]

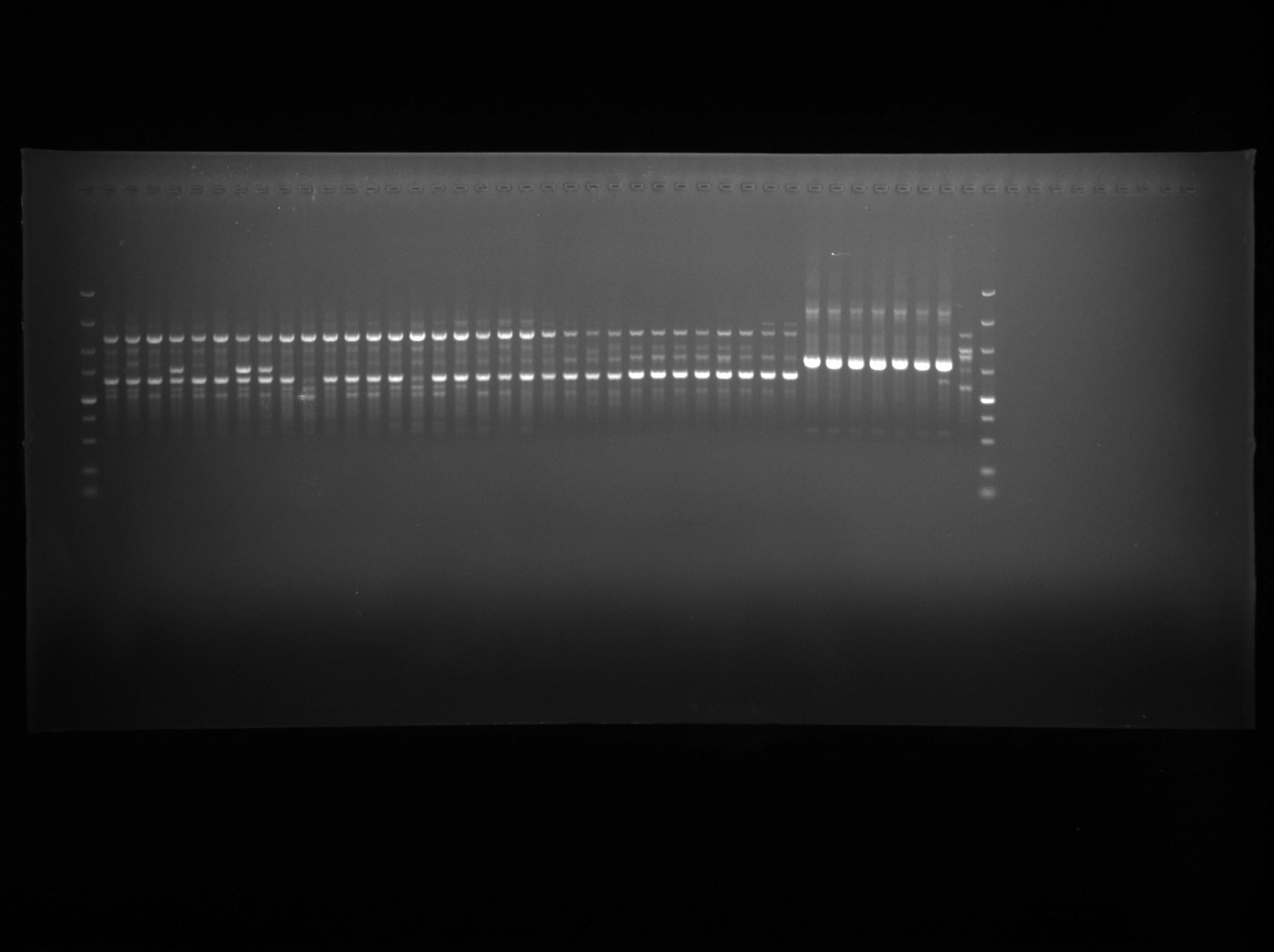

Supplement: Supplemental Information 6 — PCR banding patterns of 136 accessions of Torenia using seven primers. [file peerj-09-11702-s006.zip › iPBS_electrophoresis/Primer 2076 accessions 97-136.jpg]

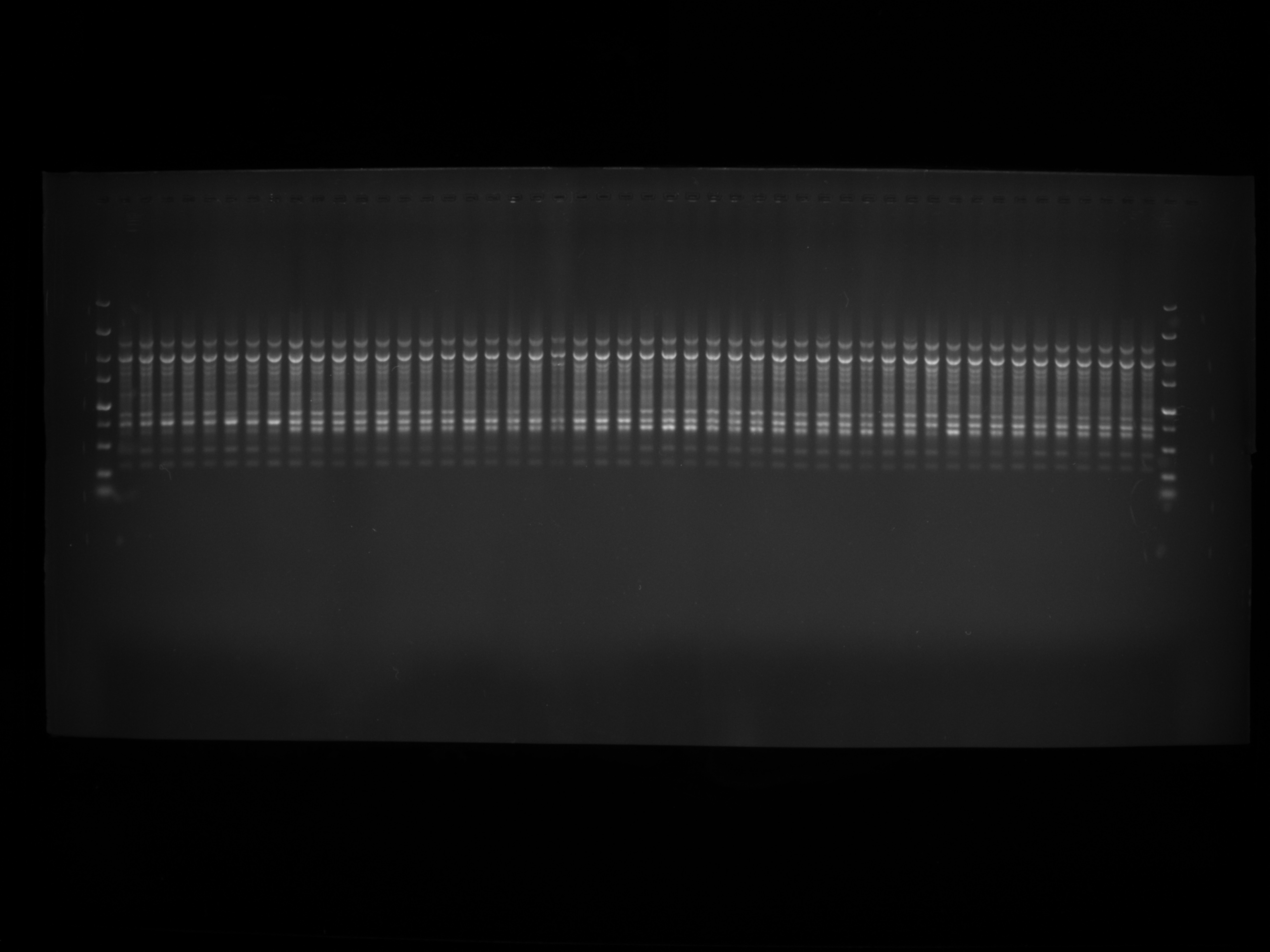

Supplement: Supplemental Information 6 — PCR banding patterns of 136 accessions of Torenia using seven primers. [file peerj-09-11702-s006.zip › iPBS_electrophoresis/Primer 2077 accessions 1-48.jpg]

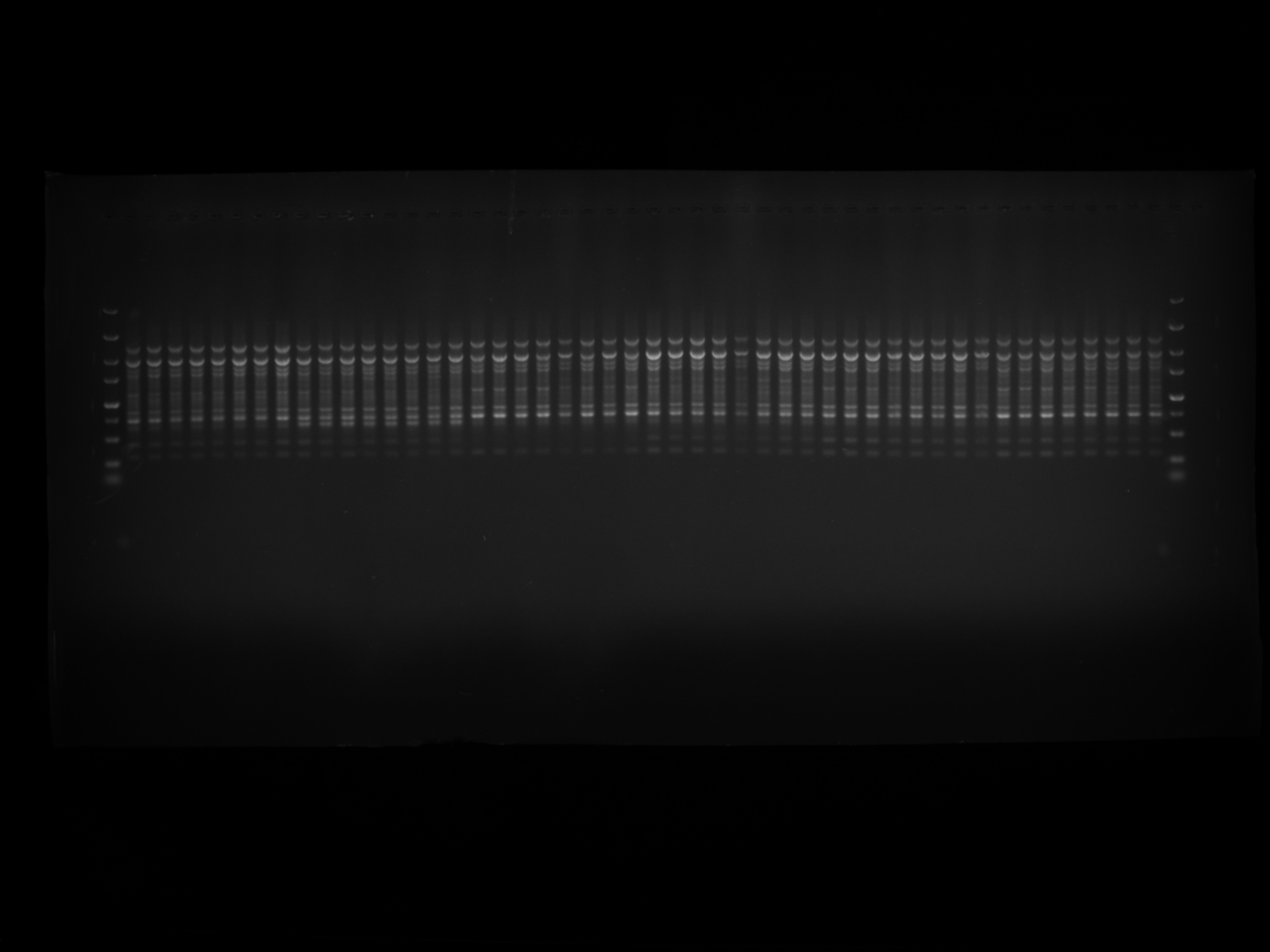

Supplement: Supplemental Information 6 — PCR banding patterns of 136 accessions of Torenia using seven primers. [file peerj-09-11702-s006.zip › iPBS_electrophoresis/Primer 2077 accessions 49-96.jpg]

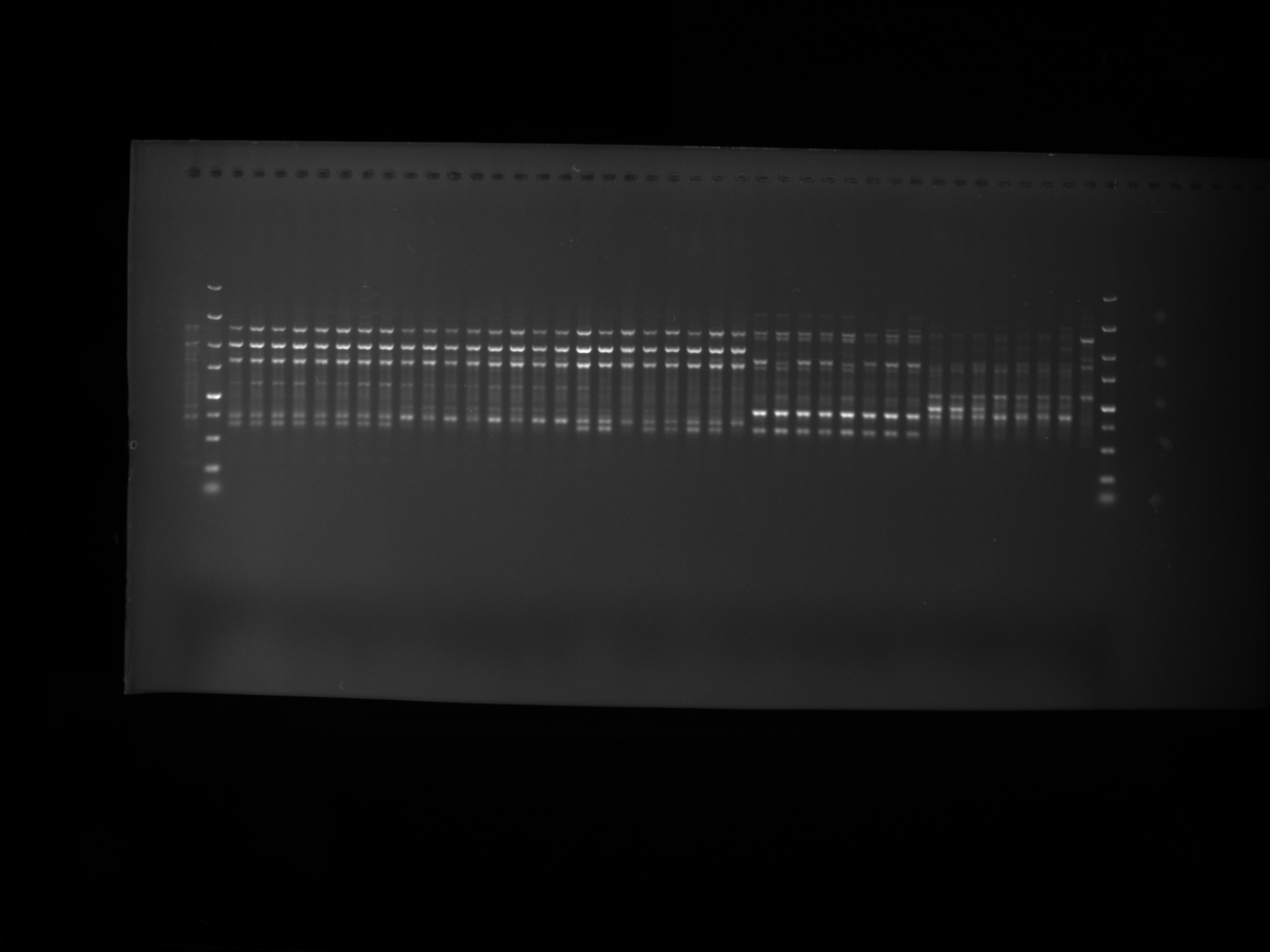

Supplement: Supplemental Information 6 — PCR banding patterns of 136 accessions of Torenia using seven primers. [file peerj-09-11702-s006.zip › iPBS_electrophoresis/Primer 2077 accessions 97-136.jpg]

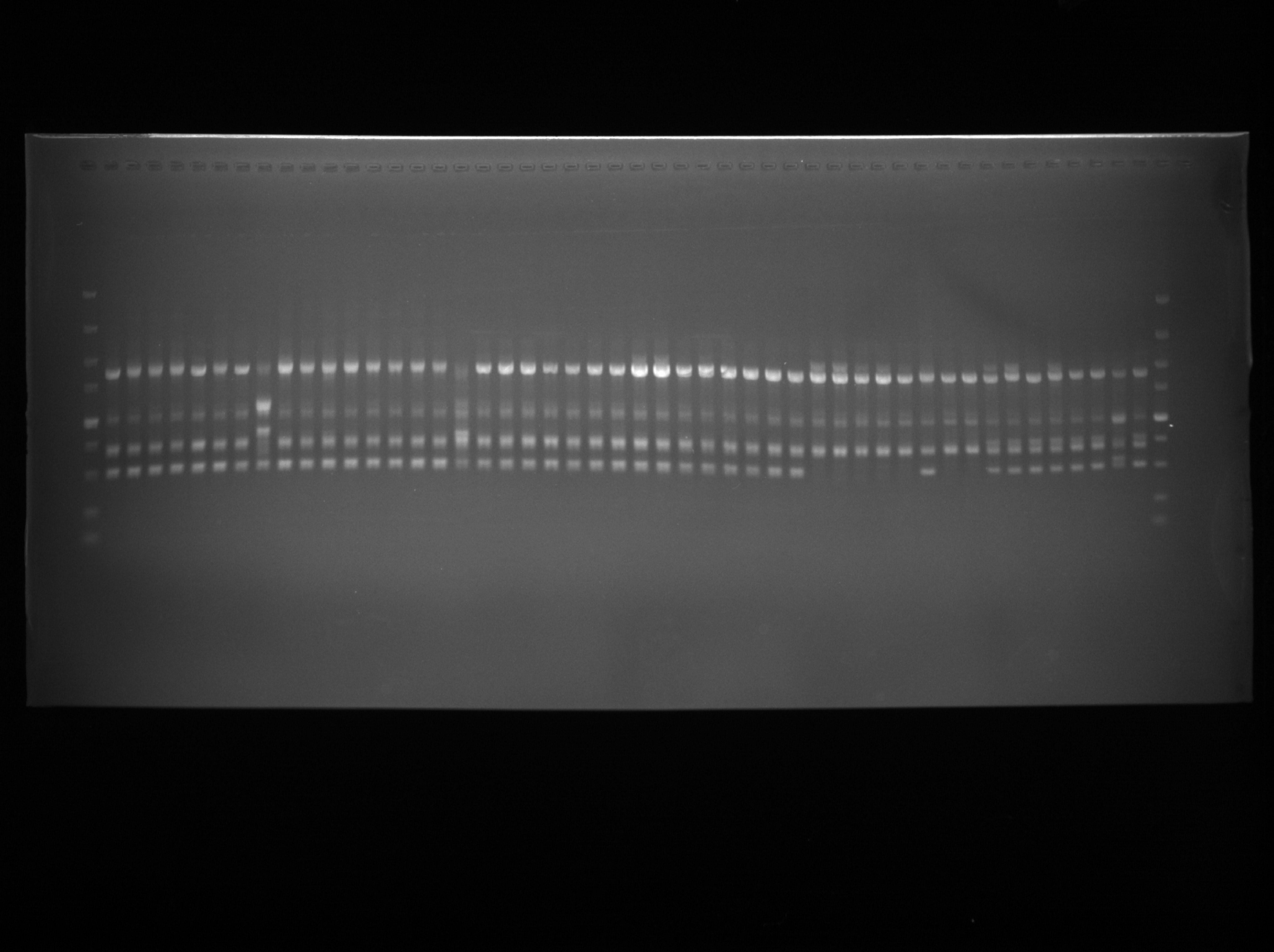

Supplement: Supplemental Information 6 — PCR banding patterns of 136 accessions of Torenia using seven primers. [file peerj-09-11702-s006.zip › iPBS_electrophoresis/Primer 2272 accessions 1-48.jpg]

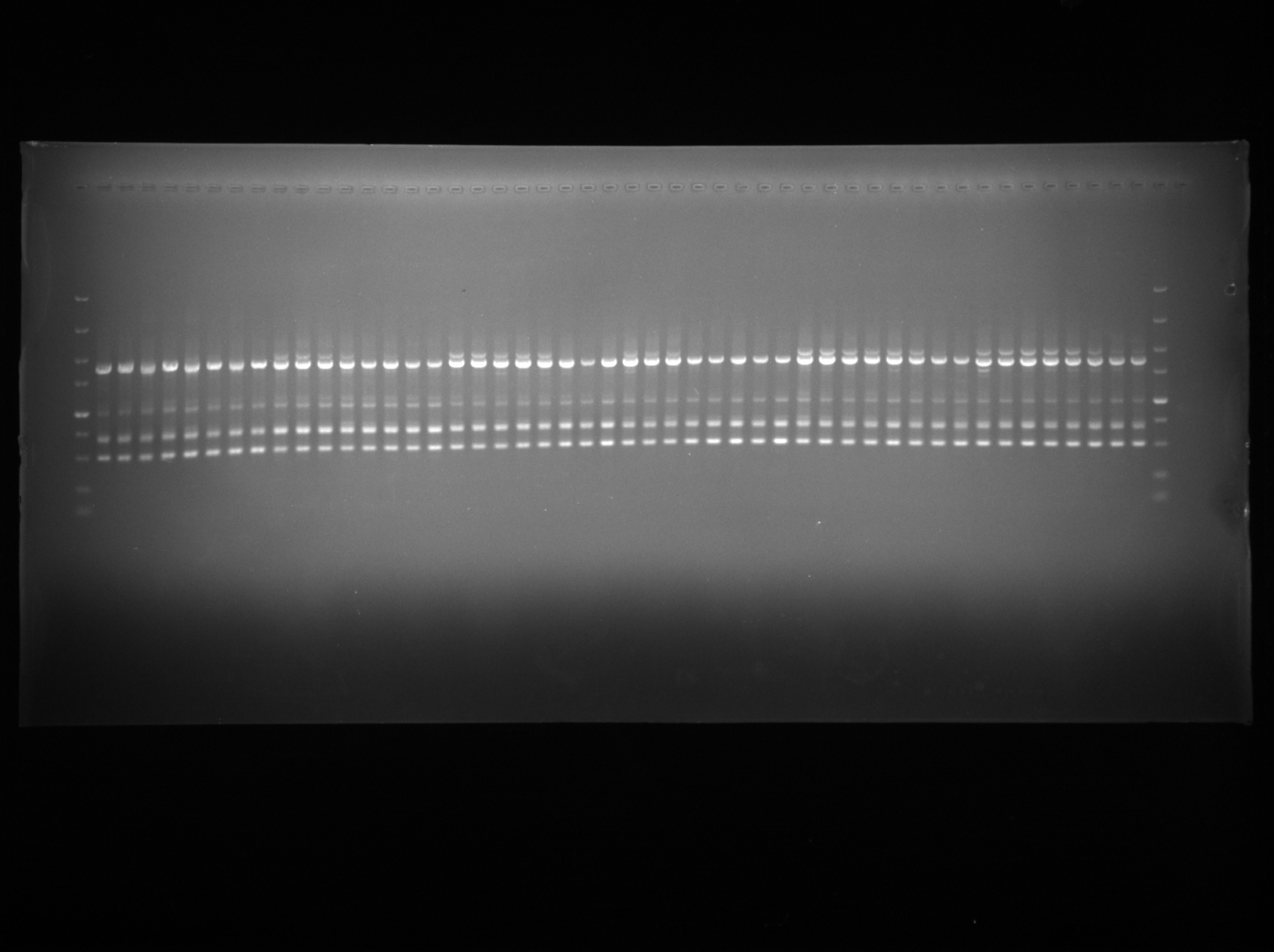

Supplement: Supplemental Information 6 — PCR banding patterns of 136 accessions of Torenia using seven primers. [file peerj-09-11702-s006.zip › iPBS_electrophoresis/Primer 2272 accessions 49-96.jpg]

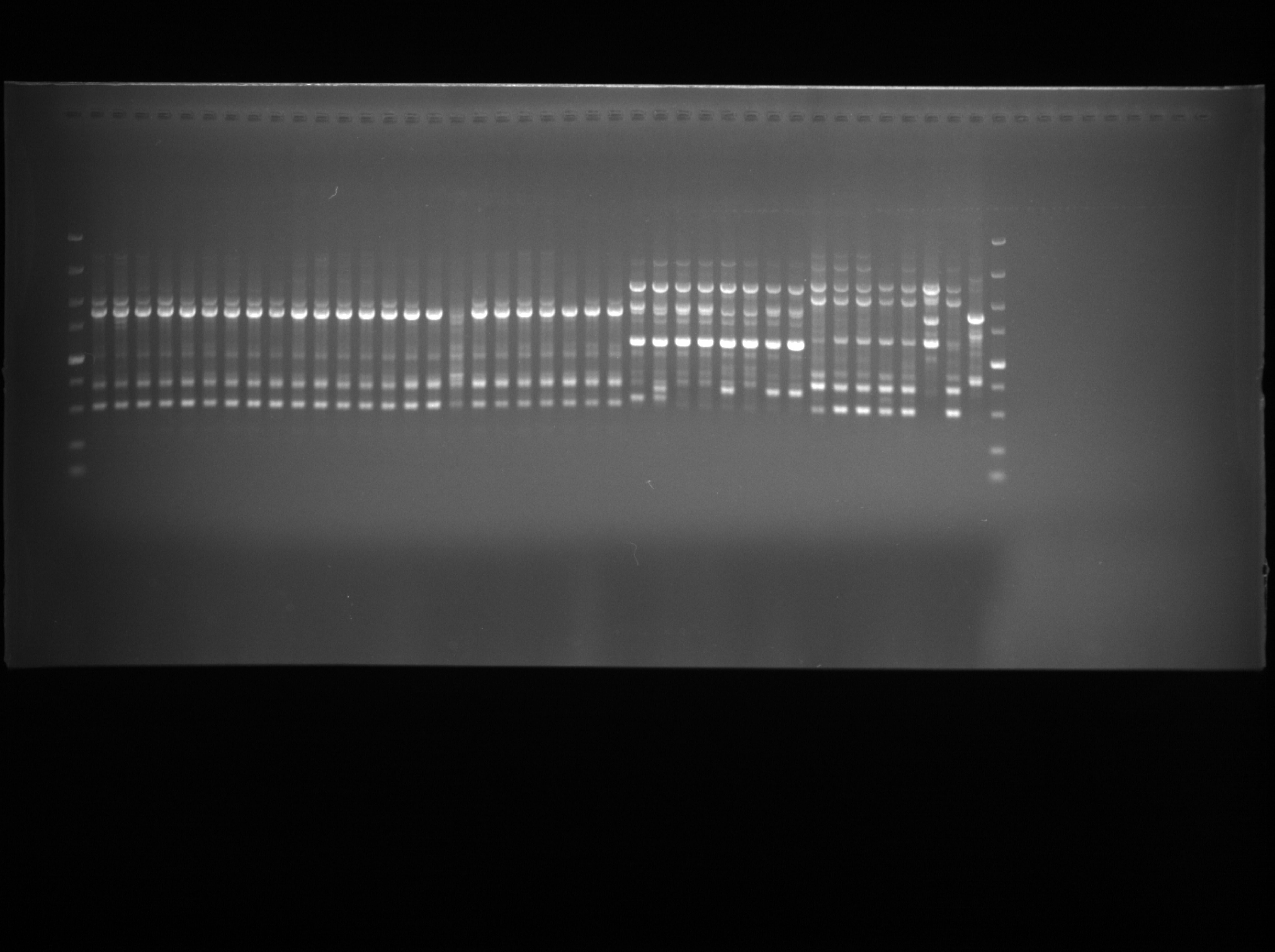

Supplement: Supplemental Information 6 — PCR banding patterns of 136 accessions of Torenia using seven primers. [file peerj-09-11702-s006.zip › iPBS_electrophoresis/Primer 2272 accessions 97-136.jpg]

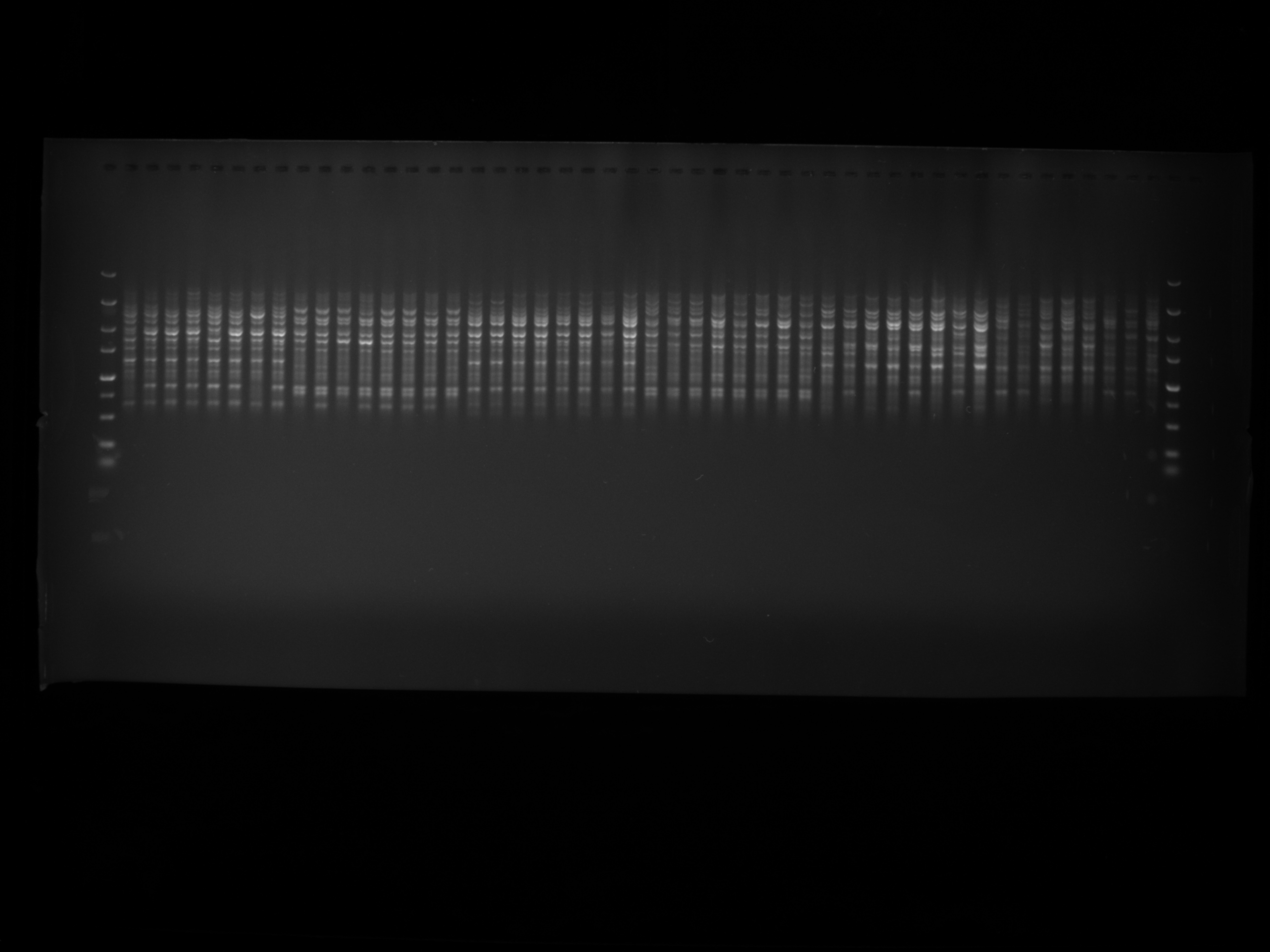

Supplement: Supplemental Information 6 — PCR banding patterns of 136 accessions of Torenia using seven primers. [file peerj-09-11702-s006.zip › iPBS_electrophoresis/Primer 2277 accessions 1-48.jpg]

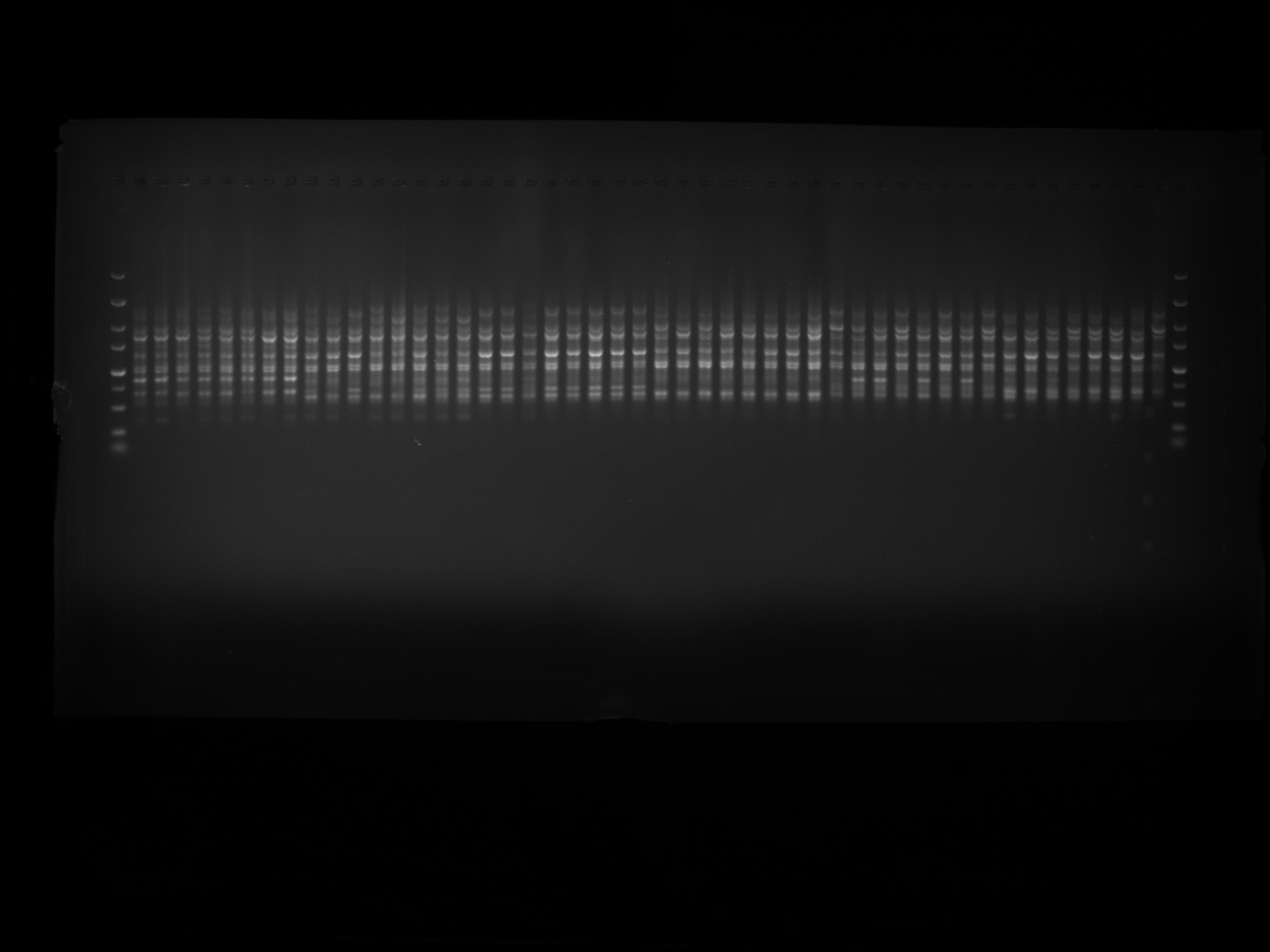

Supplement: Supplemental Information 6 — PCR banding patterns of 136 accessions of Torenia using seven primers. [file peerj-09-11702-s006.zip › iPBS_electrophoresis/Primer 2277 accessions 49-96.jpg]

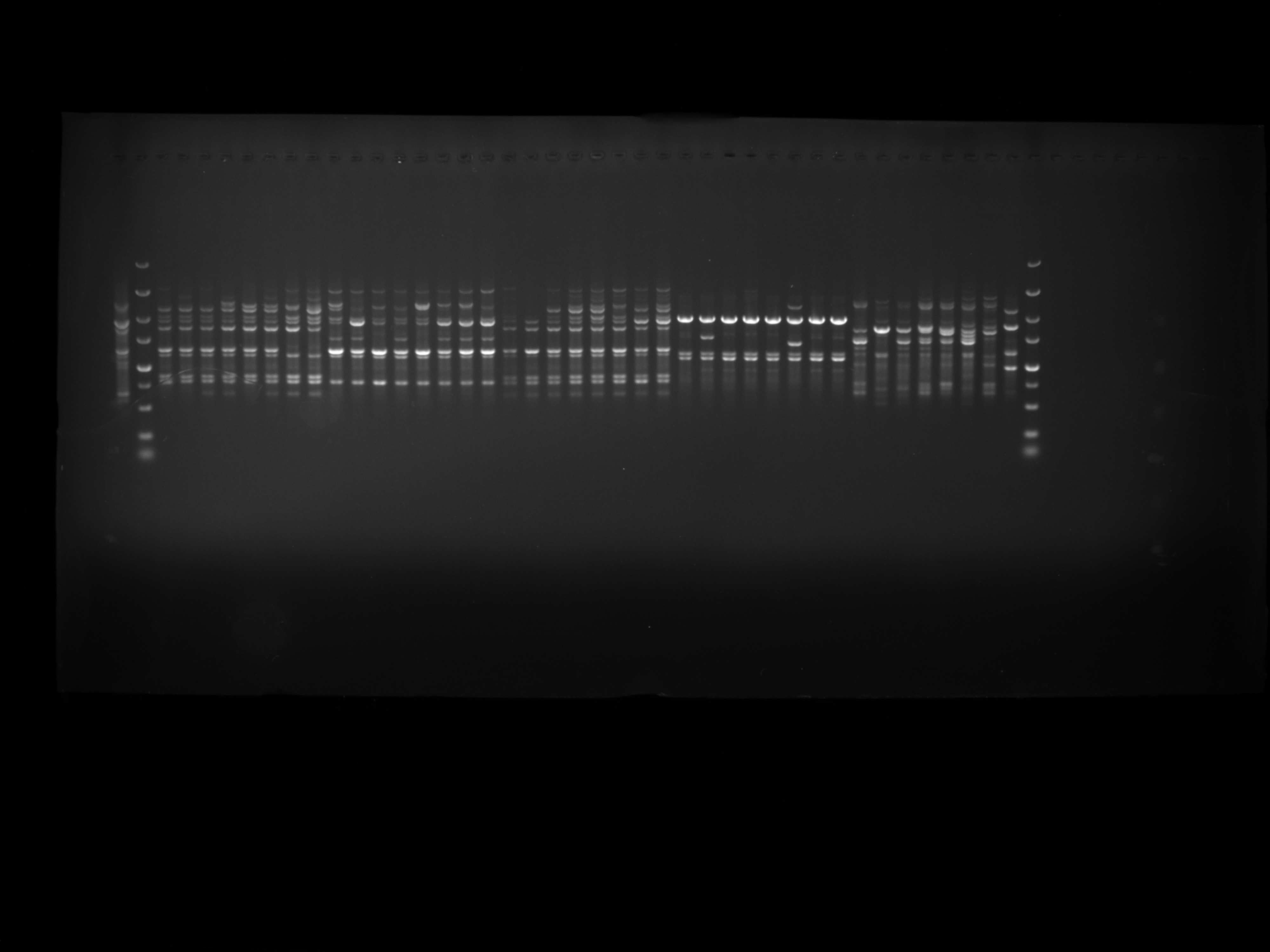

Supplement: Supplemental Information 6 — PCR banding patterns of 136 accessions of Torenia using seven primers. [file peerj-09-11702-s006.zip › iPBS_electrophoresis/Primer 2277 accessions 97-136.jpg]

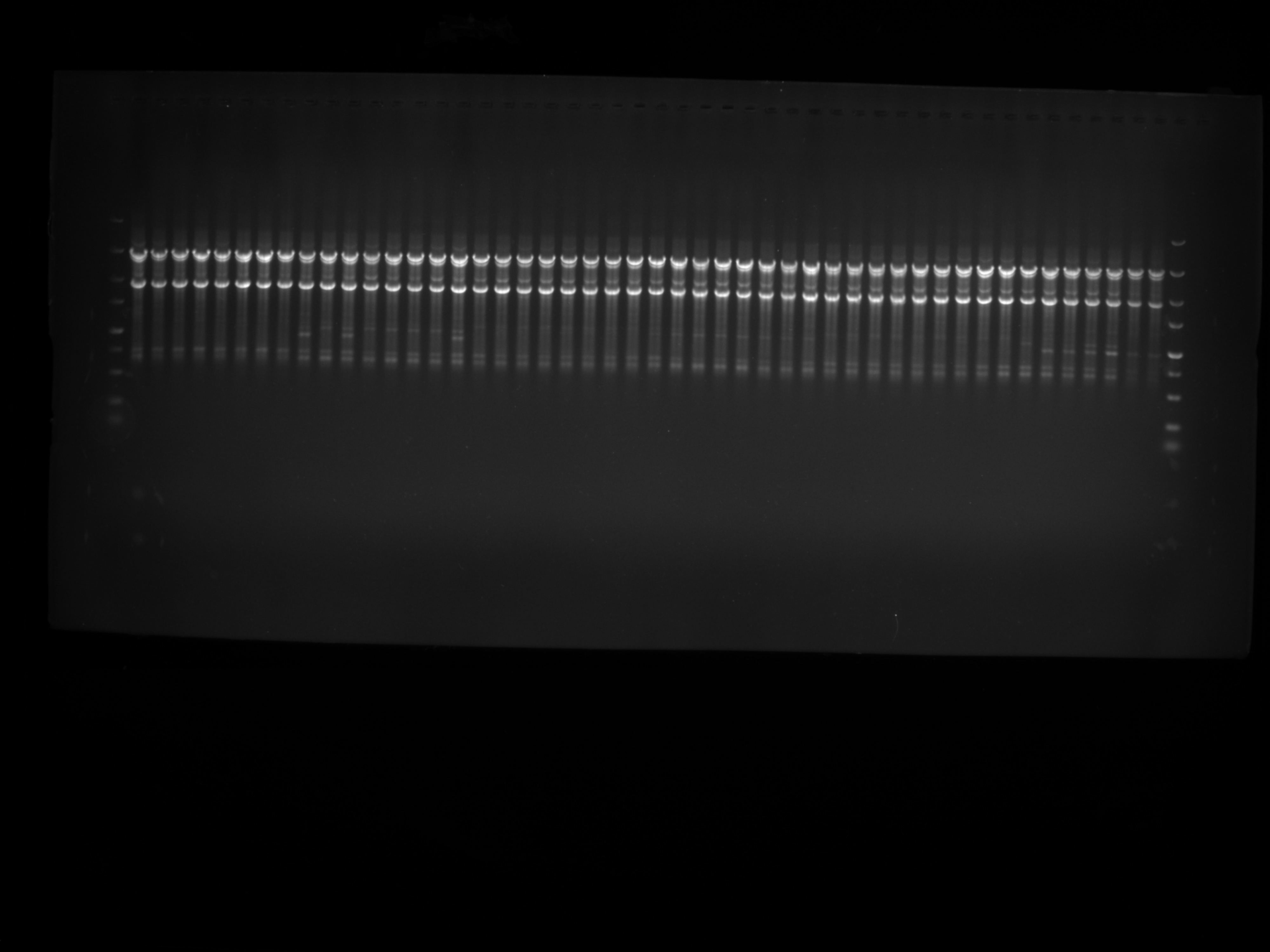

Supplement: Supplemental Information 6 — PCR banding patterns of 136 accessions of Torenia using seven primers. [file peerj-09-11702-s006.zip › iPBS_electrophoresis/Primer 2377 accessions 1-48.jpg]

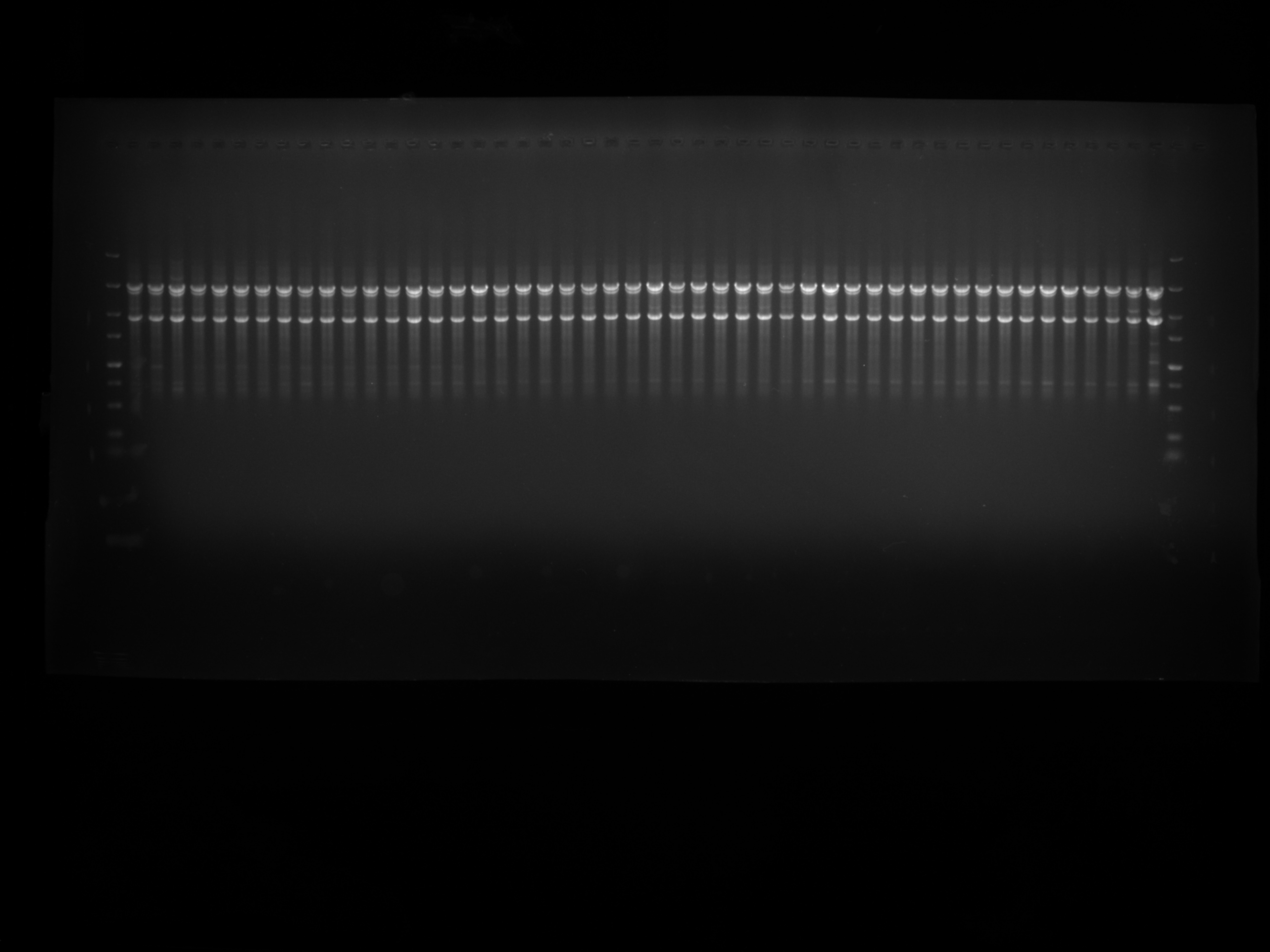

Supplement: Supplemental Information 6 — PCR banding patterns of 136 accessions of Torenia using seven primers. [file peerj-09-11702-s006.zip › iPBS_electrophoresis/Primer 2377 accessions 49-96.jpg]

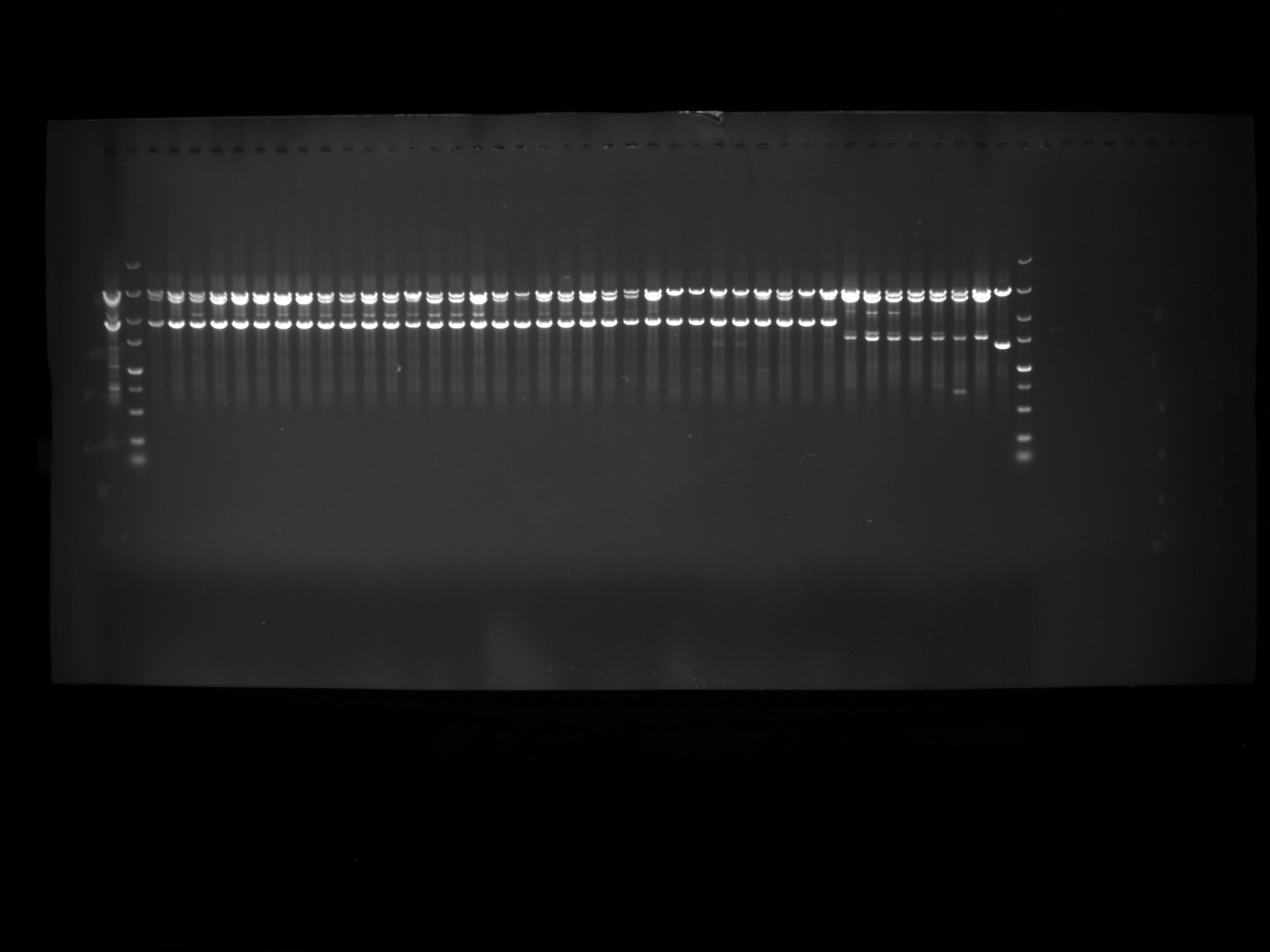

Supplement: Supplemental Information 6 — PCR banding patterns of 136 accessions of Torenia using seven primers. [file peerj-09-11702-s006.zip › iPBS_electrophoresis/Primer 2377 accessions 97-136.jpg]

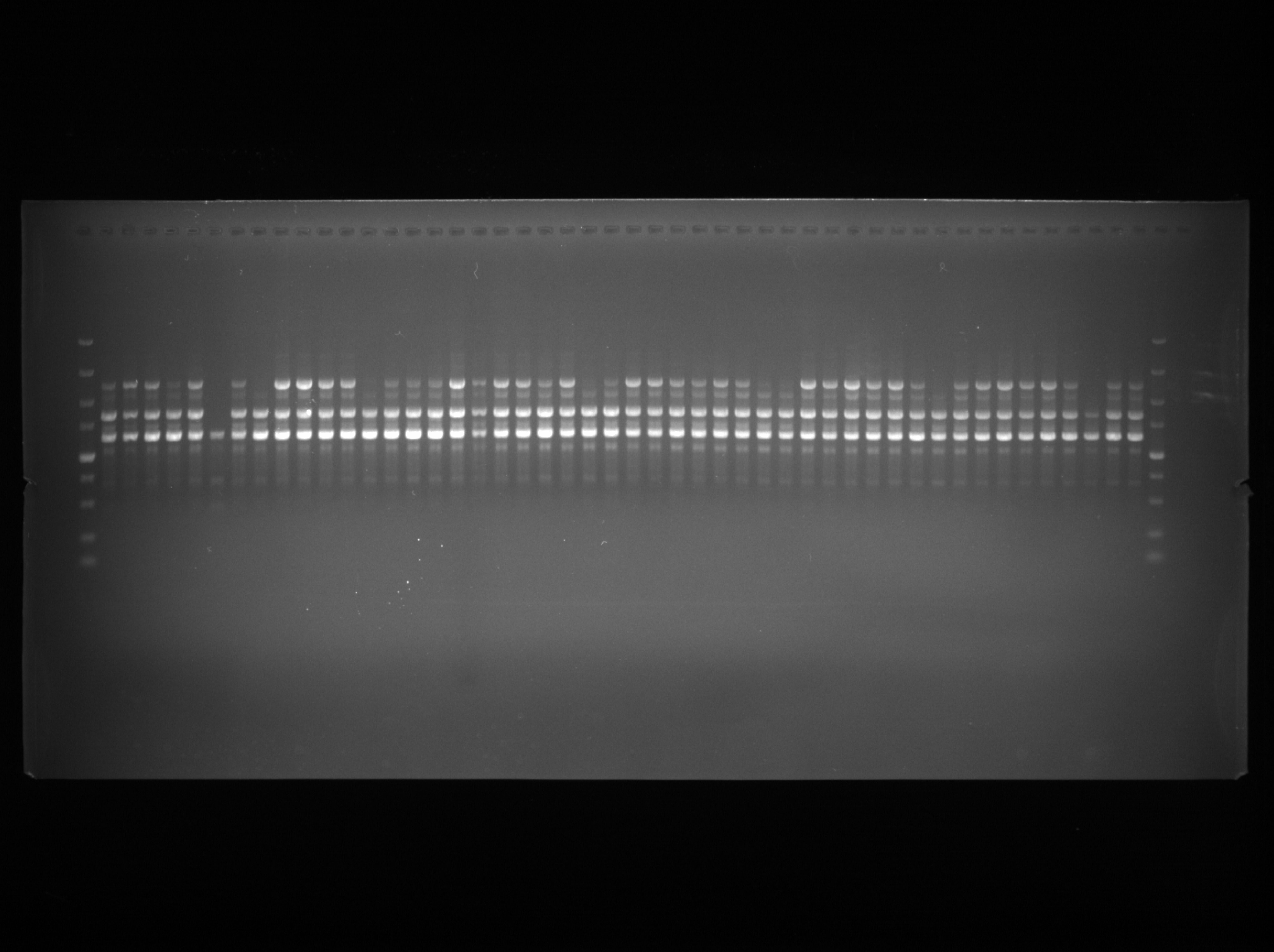

Supplement: Supplemental Information 6 — PCR banding patterns of 136 accessions of Torenia using seven primers. [file peerj-09-11702-s006.zip › iPBS_electrophoresis/Primer 2383 accessions 1-48.jpg]

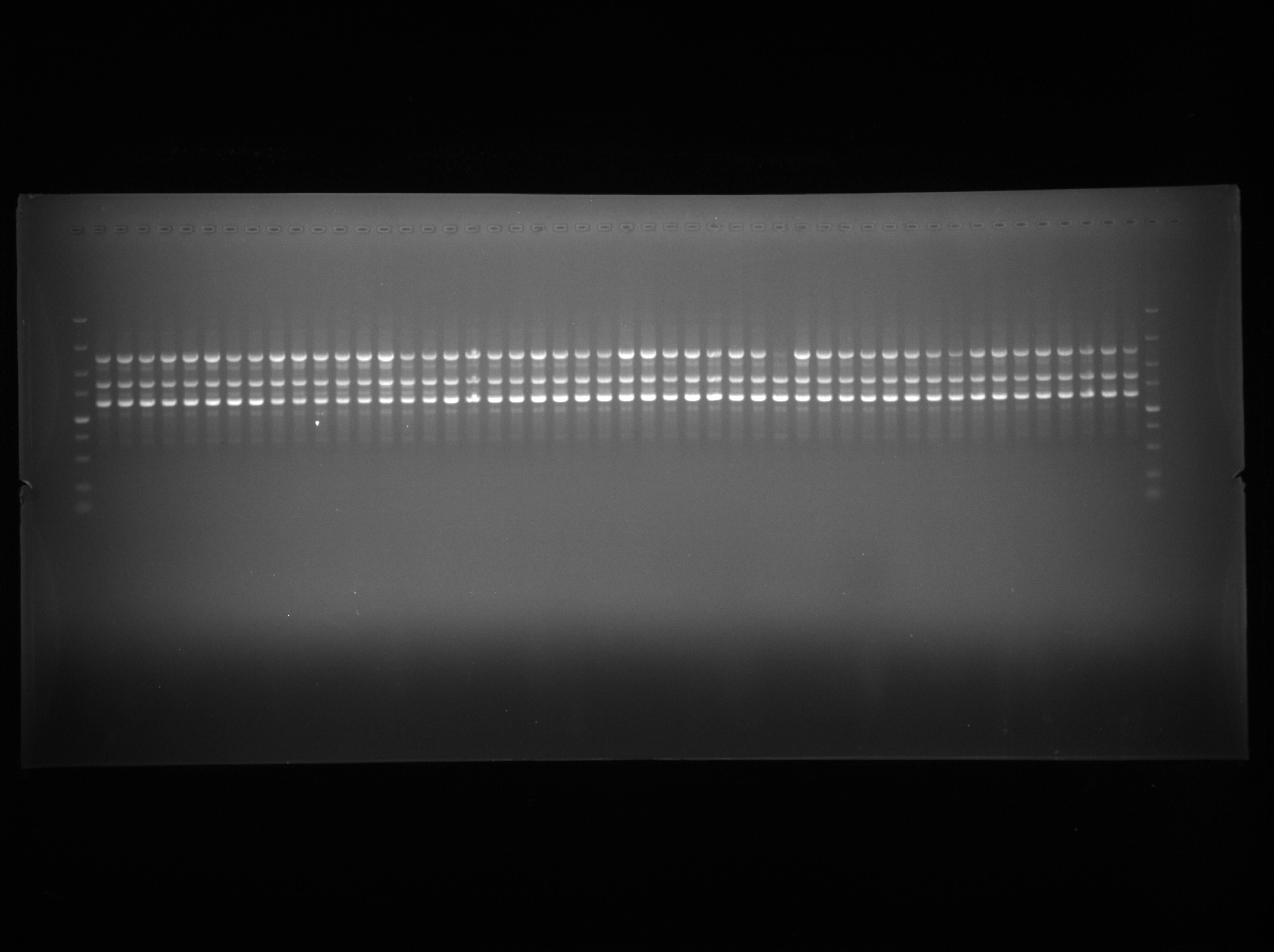

Supplement: Supplemental Information 6 — PCR banding patterns of 136 accessions of Torenia using seven primers. [file peerj-09-11702-s006.zip › iPBS_electrophoresis/Primer 2383 accessions 49-96.jpg]

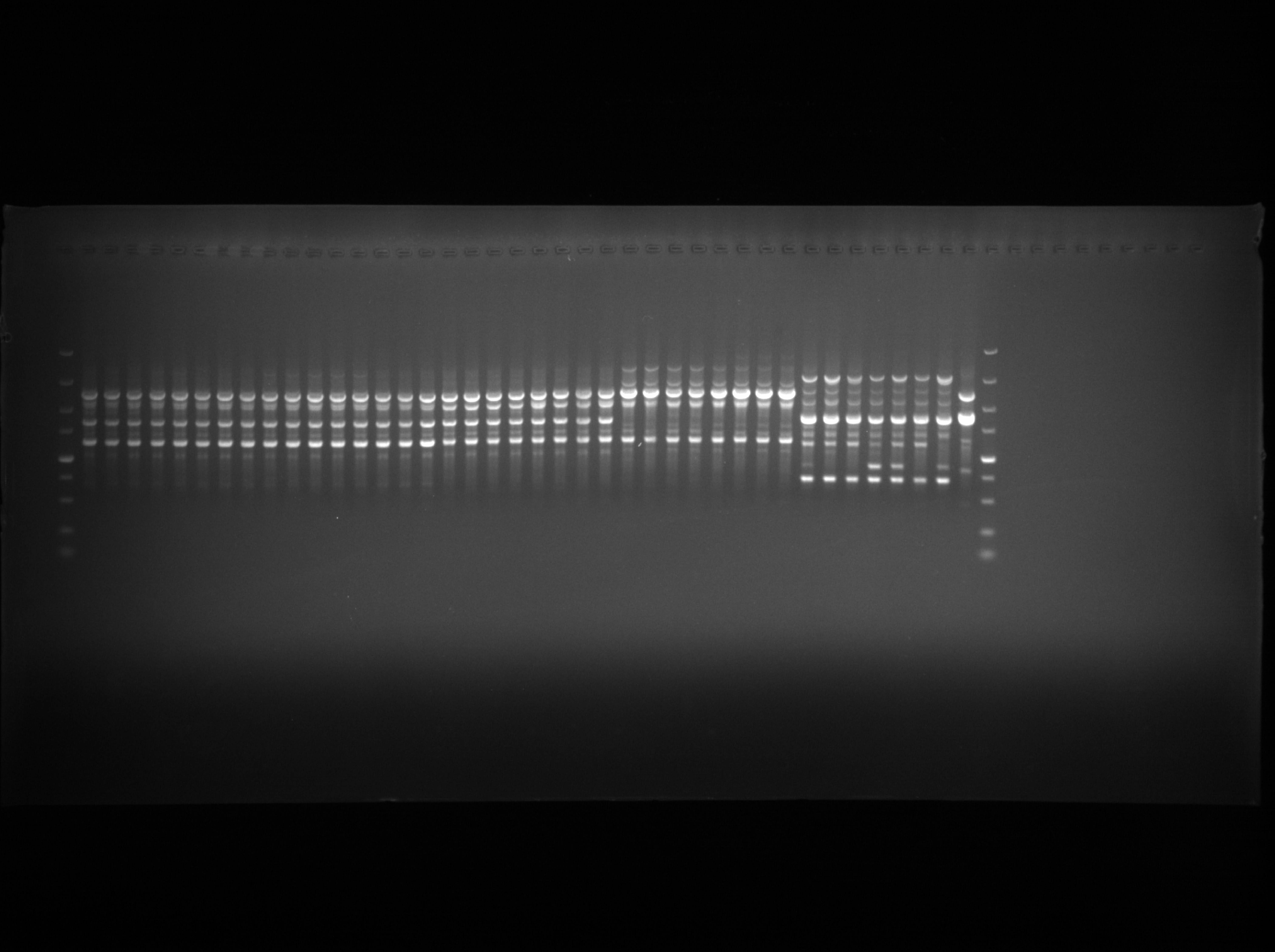

Supplement: Supplemental Information 6 — PCR banding patterns of 136 accessions of Torenia using seven primers. [file peerj-09-11702-s006.zip › iPBS_electrophoresis/Primer 2383 accessions 97-136.jpg]

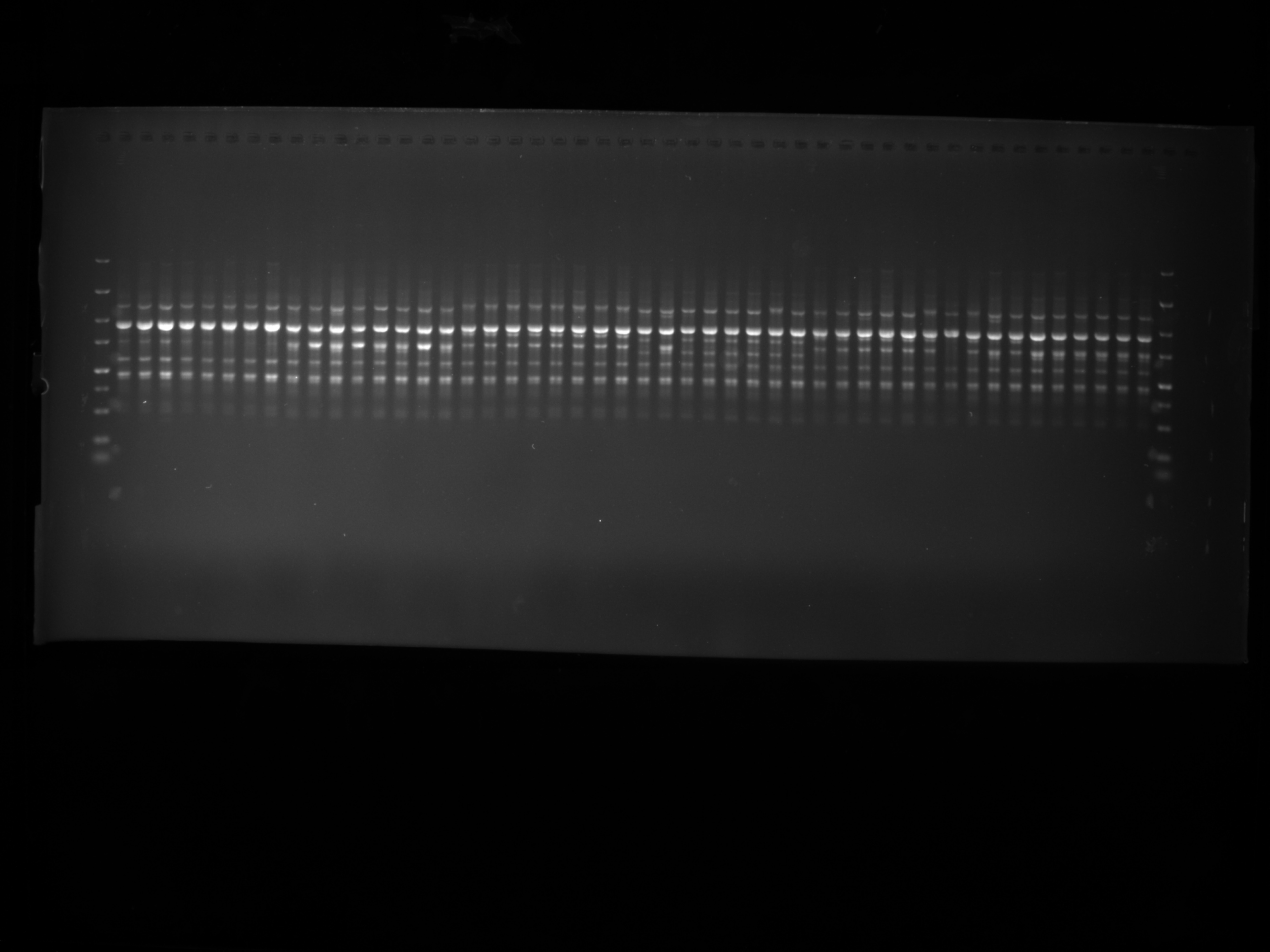

Supplement: Supplemental Information 6 — PCR banding patterns of 136 accessions of Torenia using seven primers. [file peerj-09-11702-s006.zip › iPBS_electrophoresis/Primer 2387 accessions 1-48.jpg]

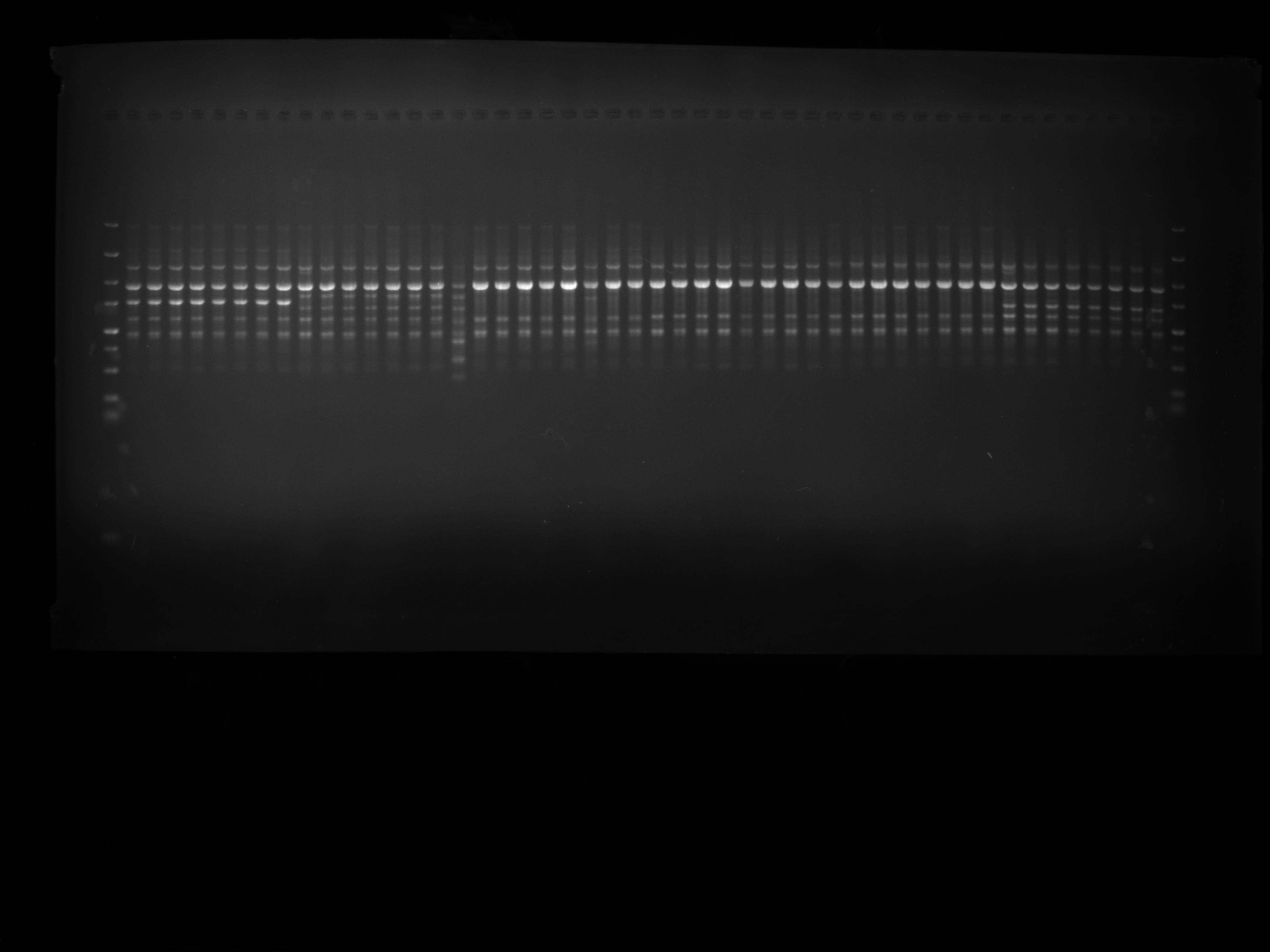

Supplement: Supplemental Information 6 — PCR banding patterns of 136 accessions of Torenia using seven primers. [file peerj-09-11702-s006.zip › iPBS_electrophoresis/Primer 2387 accessions 49-96.jpg]

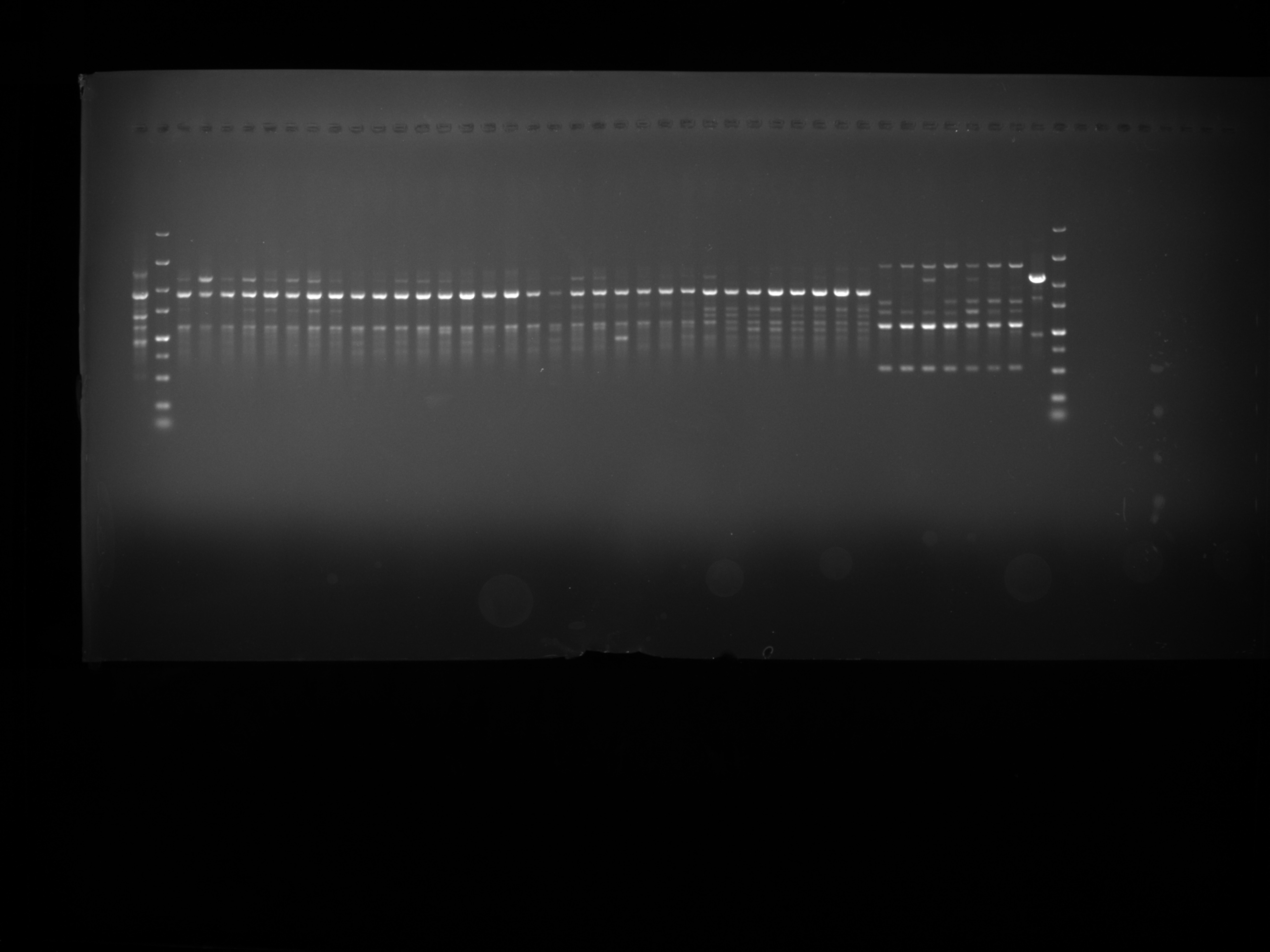

Supplement: Supplemental Information 6 — PCR banding patterns of 136 accessions of Torenia using seven primers. [file peerj-09-11702-s006.zip › iPBS_electrophoresis/Primer 2387 accessions 97-136.jpg]
